# Strategies, costs and counter‐strategies to sexual coercion

**DOI:** 10.1111/brv.70013

**Published:** 2025-04-30

**Authors:** Nikolaos Smit

**Affiliations:** ^1^ Institut des Sciences de l'Évolution, Université de Montpellier Place Eugène Bataillon Montpellier 34090 France; ^2^ Max Planck Institute for Evolutionary Anthropology Deutscher Platz 6 Leipzig 04103 Germany

**Keywords:** sexual coercion, sexual conflict, sexual harassment, female counter‐strategies, coercion costs

## Abstract

Sexual conflict, the conflict between the evolutionary interests of females and males over mating, occasionally results in the evolution of traits favourable for one sex and adverse for the other. In this context, males can use sexual coercion to increase their mating success, at the expense of their female targets' mate choice. An increasing number of studies highlight a great diversity of male and female behaviours that serve as strategies and counter‐strategies, respectively, to sexual coercion. Previous studies have reviewed the literature on infanticide but not the literature on forced copulation, sexual harassment, intimidation or punishment. This qualitative review synthesises the empirical evidence and draws a unified framework of the ecology of sexual coercion across animals, presenting male sexually coercive strategies and co‐evolved female counter‐strategies that can reduce coercion and its fitness costs. Using examples from insects to humans, it shows that different strategies of sexual coercion can lead to the evolution of similar counter‐strategies. These counter‐strategies include female promiscuity, deception of males (e.g. concealed ovulation or pseudo‐oestrus), avoidance of certain males and association with others for protection, female aggregation to dilute coercion and ultimately physical resistance by single or allied females. Extending previous work, this review provides compelling evidence of sexually antagonistic coevolution amid sexual coercion. It also calls for future work to clarify, first, which individual traits are linked to greater coercion rates in males and a higher likelihood of receiving coercion in females and, second, any causal relationships between different strategies of sexual coercion and the evolution of different social and mating systems.

## I. INTRODUCTION

The reproductive success of females and males is often limited by different factors. This may lead to sexually antagonistic coevolution that can favour the evolution of traits favourable for one sex and adverse for the other (Parker, 1979; Parker & Partridge, 1998). In anisogamous species, such sexual conflict is common in mating strategies: males commonly produce many small gametes and aim to maximise the number of their mates (Bateman, 1948) while females, which usually invest more time and energy in each reproductive event, produce few large gametes and aim to mate with one or a few ‘high‐quality’ mates (Trivers, 1972; Lessells, 2006). Three decades ago, a seminal study suggested that in this context males can use sexual coercion, i.e. force or threat of force, to increase their mating and reproductive success with female targets (direct coercion) and/or decrease the mating probability of female targets with other males (indirect coercion; Smuts & Smuts, 1993). Since then, empirical studies in ungulates (Nefdt & Thirgood, 1997), cetaceans (Scott *et al*., 2005; Connor & Vollmer, 2009), pinnipeds (Cappozzo, Túnez & Cassini, 2008), primates (Muller & Wrangham, 2009), bats (Sato, Sugiyama & Sekijima, 2023), birds (Pizzari & Birkhead, 2000), reptiles (Moldowan, Brooks & Litzgus, 2020a), amphibians (Itoh, 2023), fish (Magurran & Ojanguren, 2007), molluscs (Kimura & Chiba, 2015), insects (Singh, Singh & Mishra, 2022) and other taxa (Cassini, 2021) have highlighted the prevalence and influence of sexual coercion – and the counter‐strategies against it – on the evolution of mating systems, justifying its classification as the third form of sexual selection, along with intrasexual competition and mate choice (Smuts & Smuts, 1993; Watson‐Capps, 2009).

The recent expansion of the sexual coercion literature has assigned to terms like ‘coercion’ and ‘harassment’ a connotation of sexual or reproductive function (Zucker & Kaplan, 1981; Isvaran, 2005). Yet, these terms are sometimes misused to describe any male‐to‐female aggression which, however, does not necessarily function as sexual coercion. In order to characterise male‐to‐female aggression as sexual coercion, one needs to test its functional component, verifying whether such aggression increases the mating success of the aggressor with the target (Arnqvist & Rowe, 2005; Muller & Wrangham, 2009). Importantly, male aggression and mating might be correlated independently from sexual coercion. First, males may intervene in female–female conflicts or redirect aggression to females after male–male conflicts, and females may mate with males after such incidents in order to appease them (Smuts, 1985; Watts, Colmenares & Arnold, 2000; Muller, Kahlenberg & Wrangham, 2009b). Second, males may use mild aggression to advertise their ‘quality’ to females (Smuts & Smuts, 1993; Robbins, 2009) and females may prefer to mate with more aggressive males, for example, if these males can provide better protection services. Third, male aggression can be a by‐product of female proximity with males which is a prerequisite of mating (Soltis *et al*., 1997; Muller *et al*., 2009b; Smit *et al*., 2023). All these factors may convey a false impression of sexual coercion.

Males can also use aggression to dominate females socially (Watts & Pusey, 1993; Nishida, 2003; Muller, Kahlenberg & Wrangham, 2009a; Enigk *et al*., 2021) in contexts independent of mating, such as feeding competition (Muller *et al*., 2009b; Koenig *et al*., 2022). Some early work on human intersexual violence has even described rape as an advertisement of male power and social control (Brownmiller, 1975; Maynard & Hanmer, 1987; Stanko, 1990), although empirical studies suggest that it has sexual or reproductive motives (Ainsworth & Maner, 2012). In fact, it might be difficult to decipher the ultimate function (sexual or social/non‐sexual) of a given aggression event because social control is frequently correlated with reproductive control, that is, control over when and with whom to mate (E. Huchard. D. Lukas, N. Smit, C. Fichtel & P. M. Kappeler, in preparation), and sexual coercion appears more common in species with male‐biased social and reproductive control (Smuts, 1992; Davidian *et al*., 2022). In mandrills (*Mandrillus sphinx*; Smit *et al*., 2022a,b), chimpanzees (*Pan troglodytes*; Muller *et al*., 2007) and lions (*Panthera leo*; Bertram, 1975) males are generally larger, dominant and sexually coercive to females, while in bonobos [*Pan paniscus* (Paoli, 2009; Kappeler *et al*., 2022)], vervet monkeys [*Chlorocebus aethiops* (Andelman, 1987; Arseneau‐Robar *et al*., 2016)] and spotted hyenas [*Crocuta crocuta* (East *et al*., 2003; Strauss & Holekamp, 2019)] where females are often dominant over males and sexual size dimorphism is less pronounced, sexual coercion is negligible and male coercive attempts are usually unsuccessful. Therefore, female counter‐strategies to sexual coercion that increase female reproductive control might be linked with strategies that aim to increase female social control.

Sexual coercion was originally defined as the ‘*use by a male of force, or threat of force, that functions to increase the chances that a female will mate with him at a time when she is likely to be fertile, and to decrease the chances that she will mate with other males, at some cost to the female*’ (Smuts & Smuts, 1993, pp. 2–3). In studies of primates, this definition is commonly translated into three testable predictions: sexual coercion is male aggression that (*i*) specifically targets fertile females; (*ii*) is costly to them; and (*iii*) increases the probability of mating between the perpetrator and the female target (Muller & Wrangham, 2009; Baniel, Cowlishaw & Huchard, 2017; Kunz *et al*., 2021b; Smit *et al*., 2022a). However, studies from other taxa usually do not adopt these predictions as they do not manage to incorporate all sexually coercive strategies. Strategies like infanticide and sexual intimidation *ipso facto* (see Sections II.5 and II.3 for definitions) do not target fertile females (first prediction). Sexual intimidation and mate‐guarding might not inflict obvious measurable costs (second prediction) like injuries or stress. In fact, the restriction of female mate choice is arguably the greatest cost associated with sexual coercion aside from potential female death (McKibbin *et al*., 2008), and it might be unfeasible to measure (although recent work suggests critical female fitness consequences due to male coercion; Gómez‐Llano *et al*., 2024). Additionally, recent theoretical work suggests that female resistance to coercion can evolve to preserve the benefits of female mate choice even in the absence of direct costs of coercion, such as injuries (Snow *et al*., 2019). Finally, sexual punishment might not increase the probability of mating between the perpetrator and the female target (third prediction) but only decrease that of the target female with other males (indirect coercion).

The functional component of sexual coercion includes the enforcement of the perpetrator's mate preference at the expense of the target's mate preference. This enforcement does not necessarily entail overt, if any, perpetrator's aggression (see Section II.6 on mate‐guarding) or a temporal association between male aggression and mating (see Section II.3 on sexual intimidation). Thus, the link between the behavioural and functional component of sexual coercion can be obscure, and the documentation of sexual coercion eventually a tricky task (Smuts & Smuts, 1993; Arnqvist & Rowe, 2005; Muller & Wrangham, 2009). In principle, male behaviours which suppress female mate preferences by compelling females to mate with males that they would not mate with otherwise, or preventing females from mating with males that they would otherwise mate with, should be classified as sexual coercion. Such male behaviours may be less conspicuous than others and to date, they lack a unified framework. Previous work has revealed the extent of infanticide as a sexually selected male reproductive strategy (Agrell, Wolff & Ylönen, 1998; Ebensperger, 1998; Palombit, 2012, 2015) but the literature on other sexually coercive strategies and the co‐evolved counter‐strategies across animals has not been compiled.

The following three sections include: a review of the most common strategies of sexual coercion from insects to humans, showing that similar strategies are observed in different taxa and they can have far less conspicuous expressions than infanticide or forced copulation (Section II); the various direct and indirect costs that sexual coercion can inflict to females (Section III); and the female counter‐strategies to sexual coercion, showing that these counter‐strategies can be similar despite the coercive strategies they are opposed to (Section IV).

## II. STRATEGIES OF SEXUAL COERCION

### (1) Forced copulation and traumatic mating

Forced copulation is likely the most evident form of sexual coercion. It is observed in apes [orangutans (Mitani, 1985); humans (‘rape’; Koss, 1993; Thornhill & Palmer, 2000; Thompson, 2009)], some bats (Sato *et al*., 2023), reptiles (Olsson, 1995; Gogliath, Ribeiro & Freire, 2010) and amphibians (Roberts & Byrne, 2011) as well as various birds (Barash, 1977; McKinney & Stolen, 1982; Afton, 1985; Emlen & Wrege, 1986; Sorenson, 1994; Low, 2005; Rohwer, Harris & Walsh, 2014; Hooper *et al*., 2024) and invertebrates (Thornhill, 1980; Cordero, 1999; Cordero & Andrés, 2002; Johns *et al*., 2009). It usually involves the physical restraint of a female by one, or occasionally more, males followed by intromission/insemination despite the female's resistance. The forcible spawning of external fertilisers (e.g. some fishes; Taborsky, 2008) can have similar behaviours and function. In certain species, males have morphological traits, such as genitalic claws, that facilitate forced copulation (Kwan *et al*., 2013). In some molluscs (Chase & Blanchard, 2006; Kimura & Chiba, 2015) and arthropods (Tatarnic, Cassis & Siva‐Jothy, 2014; Reinhardt, Anthes & Lange, 2015) males can even pierce females' bodies with their reproductive organs and inject sperm that can fertilise the female target through the wound (‘traumatic mating/insemination’; Lange *et al*., 2013). Yet, forced copulation might not always involve extreme aggression levels (Bisazza, Vaccari & Pilastro, 2001) when, for example, the risks of resistance for females exceed the cost of forced copulation itself and females prefer to concede with little or no resistance. Finally, in some poecilid species, copulation is almost exclusively forced as females never cooperate (Martin, 1975; Farr, 1989; Bisazza, 1993). Although the reasons behind the latter case are not clear, it might reflect high costs of mating for females that lead them to minimise mating events.

### (2) Sexual harassment

**Fig. 1:**
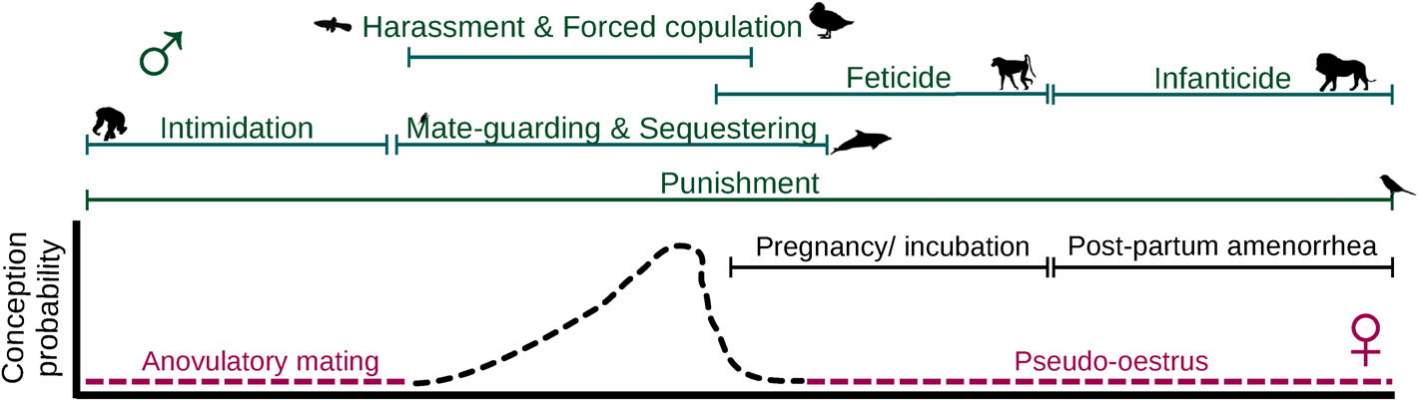
Timing of the most common strategies of sexual coercion (top/turquoise) within the female reproductive cycle (black/middle). The periods where females can deceive males by adapting the timing of mating are also shown (bottom/red).

‘Sexual harassment’ has been used to describe different male mating strategies and it is often used without being strictly defined (Cappozzo *et al*., 2008). In studies on humans, ‘sexual harassment’ is occasionally undefined (Nuñez *et al*., 2015; Ollo‐López & Nuñez, 2018; Folke *et al*., 2020) or it is loosely defined as any unwelcome ‘sexualised’ behaviour (McLaughlin, Uggen & Blackstone, 2012; Hennekam & Bennett, 2017) similar to ‘sexual abuse’ (Cense & Brackenridge, 2001) or any ‘improper’ behaviour that has a ‘sexual dimension’ (O'Donohue, Downs & Yeater, 1998). However, in other animals ‘sexual harassment’ is usually used to describe repeated male (aggressive) mating attempts which incite females to concede and mate with their harassers (Smuts & Smuts, 1993; Clutton‐Brock & Parker, 1995). Thus, it resembles forced copulation in the short‐term temporal association of male aggression with perpetrator–target mating (Fig. 1). Relevant behaviours had been reported long before the documentation of sexual coercion as a widespread array of male sexual behaviours. Fallow deer (*Dama dama*) males were reported to attempt copulation persistently with initially resisting females (Clutton‐Brock *et al*., 1988) and small tortoiseshell butterfly (*Aglais urticae*) males were reported to tap females persistently with their antennae until they drop to the ground where males might achieve mating (Baker, 1972). Sexual harassment has since been documented in an increasing number of species including other ungulates (Nefdt & Thirgood, 1997; Saunders *et al*., 2005) and insects (Rossi, Nonacs & Pitts‐Singer, 2010) as well as fishes (Magurran & Ojanguren, 2007; Dadda, 2015) and pinnipeds (Galimberti, Boitani & Marzetti, 2000b). In certain pinnipeds male harassment can lead to infant–mother separation (Campagna, Le Boeuf & Cappozzo, 1988) or interruption of lactation (Boness, Bowen & Iverson, 1995) with occasionally fatal consequences for infants and potentially similar benefits for male harassers as infanticide (see Section II.5).

### (3) Sexual intimidation

In contrast to forced copulation and sexual harassment, sexual intimidation does not presuppose a strict temporal association of male aggression and mating with the target but aims at delayed benefits (Fig. 1). Males are coercive during the pre‐receptive period to increase the probability of mating during the receptive period of the female target (Smuts & Smuts, 1993; Clutton‐Brock & Parker, 1995). Sexual intimidation has only been documented in chimpanzees (Muller *et al*., 2007), chacma baboons (*Papio ursinus*; Baniel *et al*., 2017) and mandrills (Smit *et al*., 2022a). While the studies on the first two species analysed only patterns of severe physical aggression, the study on mandrills showed that males of this species use also mild aggression, such as non‐physical threats, to intimidate females (Smit *et al*., 2022a), reminiscent of coercive threats by men (World Health Organization, 2012). This result confirms that sexual coercion does not always entail overt aggression. Importantly, the combination of such inconspicuous behaviours used by males and the temporal uncoupling of male aggression and mating may suggest that sexual intimidation occurs also in other species but has to date been overlooked [e.g. see Link, Di Fiore & Spehar (2009) for similar male‐to‐female aggression patterns in spider monkeys (genus *Ateles*)]. Ultimately, the recent documentation of sexual intimidation has led to reconsideration of previous interpretations of female behaviours. For example, female approaches or presentations (presenting one's hindquarters – a common behaviour in primates) to males were previously perceived as expression of female mate preference (Stumpf & Boesch, 2006) but they might, in fact, reflect compromise or appeasement in response to male sexual coercion (Muller *et al*., 2007; Baniel *et al*., 2021).

### (4) Sexual punishment

Sexual intimidation is occasionally associated with another sexually coercive strategy, termed sexual punishment, creating some definition inconsistencies (Clutton‐Brock & Parker, 1995; Prosen, Jaeger & Lee, 2004; Muller *et al*., 2009b). If one uses ‘sexual punishment’ to describe male aggression against a female who refuses to mate with the male in order to prevent future refusals, then it likely describes an aggressive component similar to sexual intimidation. However, ‘sexual punishment’ might describe a distinct male strategy, of indirect coercion, where males punish females who have mated with other males to prevent them from doing so in the future. The latter pattern, that is, punishment in response to mating with a rival male, has been reported in geladas (*Theropithecus gelada*; le Roux *et al*., 2013) and lesser grey shrikes (*Lanius minor*; Valera, Hoi & Krištín, 2003). A series of experiments in red‐backed salamanders, *Plethodon cinereus*, showed that males are more aggressive towards socially polyandrous than socially monogamous females (Jaeger, Gillette & Cooper, 2002) and, at the same time, females are more aggressive towards socially polygynous than socially monogamous male partners (Prosen *et al*., 2004), suggesting that strategies relevant to sexual punishment might have played an important role in the evolution of monogamy. In humans and certain birds, males can use coercion and particularly forced copulation as a punishment for infidelity, i.e. after interactions of their partners with extra‐pair males or when the risk of cuckoldry is high (Smuts, 1992; Basile, 2002; Goetz & Shackelford, 2006; McKibbin *et al*., 2008; Goetz *et al*., 2008; Camilleri & Quinsey, 2009). Sexual punishment can occasionally lead to uxoricide (wife‐killing), which is most likely a non‐adaptive collateral damage of male coercive control (but see Shackelford, Buss & Weekes‐Shackelford, 2003).

### (5) Infanticide and feticide

While the killing of females is probably maladaptive, the killing of dependent infants or fetuses is often a sexually selected coercive strategy. Infanticide can be adaptive when males kill infants sired by other (usually unrelated) males and victims' mothers resume oestrus cycling and mate with the perpetrators faster than they would mate if they had to wean their current infants first (Hrdy, 1979; van Schaik & Janson, 2000). Infanticide is common in mammals that live in groups with more females per male (high adult sex ratio) and dominant males have high reproductive skew but short tenures (Lukas & Huchard, 2014). It is particularly common in mammals, including primates (Watts, 1989; Teichroeb & Sicotte, 2008), carnivores (Bertram, 1975; Smith *et al*., 2015), and equids (Berger, 1983; Pluháček & Bartoš, 2000; Feh & Munkhtuya, 2008; Gray, 2008; but see Kirkpatrick & Turner, 1991) but it has been documented also in insects (Trumbo, 1990), birds (Crook & Shields, 1985; Freed, 1986; Veiga, 2000; Møller, 2004) and fish (Jindal *et al*., 2017). In some species, infanticidal attacks can be coalitionary. In lions, male coalitions often take over prides and kill young cubs sired by the ousted male(s) (Bertram, 1975; Packer & Pusey, 1983) and in killer whales (*Orcinus orca*), a female has been observed to assist her adult son in the killing of an unrelated infant (Towers *et al*., 2018).

Male‐induced fetal loss can likely offer similar benefits of infanticide to male perpetrators. Two different phenomena have been documented where fetal loss is male induced (Zipple *et al*., 2019). First, similarly to infanticide, non‐sire males can commit feticide using aggression towards a female resulting in the termination of an established pregnancy either due to the target's stress response or by directly harming the fetus (Pereira, 1983; Agoramoorthy *et al*., 1988; Zipple *et al*., 2017). Second, in some species of rodents (Bruce, 1959; Parkes & Bruce, 1961; Stehn & Richmond, 1975; Hackländer & Arnold, 1999), primates (Roberts *et al*., 2012; Amann, Pines & Swedell, 2017) and other mammals (Bartoš *et al*., 2011, 2016), the costs of infanticide and feticide have likely promoted the evolution of female physiological mechanisms of premature pregnancy termination (‘Bruce effect’). It usually occurs after exposure of pregnant females to non‐sire males with no need for male aggression and although it is not a behavioural male strategy and sexual coercion *sensu stricto*, from the male perspective, it can offer similar mating and reproduction benefits to infanticide and feticide, while from the female perspective, it can reduce the costs of potential future infanticide.

### (6) Mate‐guarding and sequestering

In contrast to most sexually coercive strategies that involve a critical component of male aggression, mate‐guarding is a widespread male reproductive strategy [insects (Baxter, Barnett & Dukas, 2015); amphibians (Rueda‐Solano *et al*., 2022); ungulates (Poole, 1989), including cetaceans (Willis & Dill, 2007); primates (Setchell, Charpentier & Wickings, 2005); pinnipeds (McRae & Kovacs, 1994); rodents (Waterman, Wolff & Sherman, 2007); Macroscelidea/shrews (Gibbon, 1997); crustaceans (Cothran, 2020)] that does not necessarily involve aggression but can still, occasionally, qualify as direct or indirect sexual coercion (Muller & Wrangham, 2009). Males may guard females during oestrus periods to prevent approaches and mating attempts of rival males (indirect coercion) or post‐mating when they aim to ensure fertilisation of the guarded female [tammar wallabies, *Macropus eugenii* (Rudd, 1994); rove beetles, *Leistotrophus versicolor* (Alcock & Forsyth, 1988)] or fertilisation of a greater proportion of the female's eggs [various insects (Parker, 1970; Alcock, 1994); direct coercion]. Importantly, its intensity might depend on social context or demographic changes, increasing, for example, during or shortly after male take‐overs [hamadryas baboons, *Papio hamadryas hamadryas* (Swedell & Schreier, 2009)] or in the presence of rival males [various primates (Cooper, Aureli & Singh, 2004; Robbins & Sawyer, 2007)].

Males that exercise mate‐guarding might be aggressive towards the guarded females, rival males, both, or none (Birkhead, Johnson & Nettleship, 1985; Poole, 1989; Nishida, 1990; Alberts, Altmann & Wilson, 1996; Foitzik *et al*., 2002; Setchell *et al*., 2005; Willis & Dill, 2007; Baxter *et al*., 2015; Rueda‐Solano *et al*., 2022). Previous work suggests that mate‐guarding with no male‐to‐female aggression is unlikely to be coercive, but instead it might reflect that females are amenable to being guarded (Muller *et al*., 2009b). Indeed, mate‐guarding may not qualify as sexual coercion when preferred males guard/protect females against other, undesired, males (Brereton, 1995; Harcourt & Greenberg, 2001). However, mate‐guarding may occasionally reflect sexual coercion even in the absence of male‐to‐female aggression. In humans and various monogamous birds, mate‐guarding is often correlated positively with in‐pair copulation, and likely acts as an anti‐cuckoldry tactic (Shackelford *et al*., 2006). In the sexually cannibalistic praying mantid *Tenodera angustipennis*, mating delays female re‐mating with other males (Nishino *et al*., 2024). Male barn swallows (*Hirundo rustica*) use deceptive alarm calls to disrupt extrapair copulation attempts of their mates (Møller, 1990). In mandrills, where females are mate‐guarded by males on average three to four times heavier than them (Setchell *et al*., 2001, 2005), they might not actively resist male mate‐guarding, but they are observed to copulate sneakily with other males when they are out of sight of the mate‐guarding male (Smit, 2022). These examples suggest that mate‐guarding may act to restrict female mate choice even in the absence of male aggression (or female resistance) and, therefore, it may qualify as sexual coercion.

Sequestering is a similar male mating strategy to mate‐guarding involving male (threat of) force that keeps females away from their social group and, thus, from mating opportunities with other males. The term has been used to describe behaviours of bottlenose dolphins (*Tursiops* sp.; Connor, Smolker & Richards, 1992) and chimpanzees (Watts, 1998), but males in other species, including humans (Daly & Wilson, 1988; Smuts, 1992; Wilson & Daly, 2009), forcibly control the movements of females in a similar manner. For example, male Asiatic lions use aggression to prevent females from leaving their consortships and return to the core of their prides towards the end of mating events, likely increasing their relative mating success with these females (Chakrabarti & Jhala, 2019).

### (7) Other strategies

This list of sexually coercive strategies is not exhaustive. First, certain known male adaptations that maximise the chances of fertilisation and prevent female re‐mating with other males are usually not described as sexual coercion because they are based on morphological traits. Yet, these traits are strictly linked to specific male behaviours. For example, males can use penile spines to remove sperm of other males from female reproductive tracts (Waage, 1979; Eberhard, 1985; Arnqvist & Rowe, 2005) or preclude the mating of females with other males through exaggerated copulatory stimulation of females, the damage/mutilation of female genitalia (Johnstone & Keller, 2000; Stockley, 2002; Mouginot *et al*., 2015; Nakata, 2016) or the use of copulatory plugs [insects (Takami *et al*., 2008); Arachnida (Herberstein *et al*., 2012); reptiles (Moreira & Birkhead, 2004); primates (Dixson & Anderson, 2002); rodents (Dean, 2013)]. The behavioural component of these strategies might ultimately qualify as sexual coercion, as long as it restricts female mate choice. Second, recent work has documented rarer, species‐specific, strategies of sexual coercion (Link *et al*., 2009; Bro‐Jørgensen & Pangle, 2010). Males of a variety of insects may prevent or delay female mating with other males using seminal receptivity inhibitors (Holland & Rice, 1998; Arnqvist & Nilsson, 2000) anti‐aphrodisiacs (Andersson, Borg‐Karlson & Wiklund, 2000) or seminal toxins [Lung *et al*., 2002; see Arnqvist & Rowe (2005) for more examples and female counter‐strategies]. In the cannibalistic springbok mantis *Miomantis caffra*, males and females engage in aggressive wrestling, with a female victory implying consumption of the male, while a male victory implies mating (Burke & Holwell, 2021). Given the fierce female resistance and risk of injury, this mating is most likely undesired (Burke & Holwell, 2021). Male garter snakes (*Thamnophis sirtalis*) take advantage of the physiology and respiratory anatomy of females to stimulate cloacal gaping and inseminate females forcibly (Shine, Langkilde & Mason, 2003). Blue‐lined octopus (*Hapalochlaena fasciata*) males envenomate females to immobilize them and achieve copulation (Chung *et al*., 2025). [Correction added on 19 May 2025, after first online publication: The reference information of Chung *et al*. (2025) has been corrected.] In humans, the elaboration of language and technology has allowed the development of verbal (DeGue & DiLillo, 2005) and reputational [non‐consensual pornography (Englander, 2015; Walker & Sleath, 2017)] coercive tactics. However, the sexual, let alone the reproductive, function of these behaviours remains unexplored. Finally, some paradigms of so‐called ‘sex role reversal’ suggest that females can occasionally use sexual coercion as well. Similar to feticide, female *Lethocerus* water bugs can destroy non‐filial eggs despite the defence of guarding males and then mate with these males, who will subsequently guard the clutch of the destructive females (Ichikawa, 1990).

## III. COSTS OF SEXUAL COERCION FOR FEMALES

Quantitative paternity data that can be used to assess the reproductive success of male coercers are challenging to obtain [but see Feldblum *et al*. (2014) and Stieglitz *et al*. (2018) for two examples in chimpanzees and human hunter–gatherers, respectively]. Therefore, researchers usually measure the benefits of sexual coercion through male mating success with the targets of coercion (Baniel *et al*., 2017; Smit *et al*., 2022a). The impact of sexual coercion on female reproductive success is even harder to quantify (Clarke *et al*., 2009; Muller *et al*., 2009b), because of (*i*) the difficulty in assessing what a female's mating preference would be in the absence of coercion; (*ii*) the uncertainty regarding whether certain behaviours reflect mate preferences or appeasement/compromise to male coercers; and (*iii*) the longitudinal data needed to measure female reproductive success, especially in species with slow life histories. However, recent studies have documented costs of sexual coercion including both direct physical harm and indirect detrimental effects (Arnqvist & Rowe, 2005; Cassini, 2021; Gómez‐Llano *et al*., 2024) that may disrupt female reproductive cycles. The indirect costs of coercion include lower fitness due to consequences on female longevity (Dukas & Jongsma, 2012), reproductive pace (Manguette *et al*., 2019), fecundity (McLain & Pratt, 1999; Kimura & Chiba, 2015; Gómez‐Llano *et al*., 2024), offspring number (Gómez‐Llano *et al*., 2024) or offspring ‘genetic quality’ [due to increased inbreeding (Frère *et al*., 2010) or mating with undesired ‘low‐quality’ males]. These consequences on female mate choice and fitness can even lead to population decline (Kokko & Brooks, 2003; Le Galliard *et al*., 2005; Gómez‐Llano *et al*., 2024).

In conspicuous sexually coercive strategies such as infanticide, females face obvious direct costs such as injuries (Packer, Scheel & Pusey, 1990), death of their infants (Lukas & Huchard, 2014) or their own death when they try to protect their infants (Singh *et al*., 2014). An increased number of studies indicate that other sexually coercive strategies can inflict similar costs. Traumatic mating in molluscs (Chase & Blanchard, 2006; Kimura & Chiba, 2015) and various arthropods (Tatarnic *et al*., 2014; Reinhardt *et al*., 2015) can increase perpetrators' fitness by ensuring insemination through the wound, but decrease the fitness of the targets by inflicting direct costs related to wound infection, healing and reduced longevity (Lange *et al*., 2013). Increased injuries are also seen in females who face forced copulation in Lake Eyre dragons (*Ctenophorus maculosus*; Olsson, 1995) and waterfowl (McKinney & Evarts, 1998), sexual intimidation in chacma baboons (Baniel *et al*., 2017), sexual harassment in Hermann's tortoises (*Testudo hermanni*; Golubović *et al*., 2018) and certain pinnipeds (Boeuf & Mesnick, 1991; Hiruki *et al*., 1993), and intimate partner violence in humans (Novak & Hatch, 2009).

Injuries inflicted to females by male sexually coercive attempts may increase female mortality (Davies & Halliday, 1979; Clutton‐Brock & Parker, 1995). Indeed, sexual harassment can result in female death in feral sheep (*Ovis aries*; Réale, Boussès & Chapuis, 1996), eastern gray squirrels (*Sciurus carolinens*; John, 1993), sea otters (*Enhydra lutris nereis*; Staedler & Riedman, 1993), red deer (*Cervus elaphus*; Clutton‐Brock & Parker, 1995) and European common frogs (*Rana temporaria*; Dittrich & Rödel, 2023). Similarly, forced copulation can result in female death in fruit flies (*Drosophila melanogaster*; Dukas & Jongsma, 2012) and humans (Shackelford, 2002). Notably, such fatal incidents are unlikely to be adaptive since they deprive males of reproductive opportunities, besides the obvious costs for females.

Sexual coercion can increase female mortality also through its influence on predation (Rowe, 1994; Zeiss, Martens & Rolff, 1999; Shine, O'Connor & Mason, 2000). First, male coercive attempts and movements around females can attract predators (Olsson, 1995). Second, sexually harassed females might select otherwise undesirable habitats to avoid male presence, trading reduced sexual harassment with increased predation risk (Darden & Croft, 2008). Finally, coercive mating may increase female mortality due to the transmission of sexually transmitted diseases (Kalichman *et al*., 1998; Nunn, 2003; Lange *et al*., 2013).

Energetic costs are less obvious than injuries or death but they are common for females that face sexual coercion. Prolonged copulation bouts might bear high energetic costs (Robertson, 1985) and the energy needed to escape coercive males might exceed the cost of coercion itself (Watson, Stallmann & Arnqvist, 1998). Relevant costs might be imposed by males also to dependent infants of female targets, which need to keep up with their mothers and avoid (infanticidal) males. For example, in humpback whales (*Megaptera novaeangliae*), females and infants in association with multiple males show increased energy expenditure (Cartwright & Sullivan, 2009).

Male coercion can also reduce female foraging efficiency (Pilastro, Benetton & Bisazza, 2003; Plath, Parzefall & Schlupp, 2003) which can, in turn, result in unmet energetic needs [see also the distinction between maternal and fecundity costs by Cassini (2021)]. Sexual harassment in insects and fish can interrupt feeding (Odendaal, Turchin & Stermitz, 1989; Stone, 1995; Magurran & Seghers, 1997) and decrease female reproductive success (Magurran & Ojanguren, 2007; Gay *et al*., 2009; den Hollander & Gwynne, 2009; Rossi *et al*., 2010; Takahashi & Watanabe, 2010). Similarly, male coercion can interrupt female feeding and impose energetic costs on female mammals fleeing from coercive males (Berger, 1986; Rubenstein, 1986; Alberts *et al*., 1996): male bottlenose dolphins sequester females to undesirable habitats (Darden *et al*., 2009; Wallen *et al*., 2016), male chimpanzees at Ngogo interrupt cycling female feeding bouts more than twice per hour (Watts, 2022) and Bornean orangutan (*Pongo pygmaeus wurmbii*) females that associate with males have a lower feeding‐to‐moving ratio than other females (Kunz *et al*., 2021a). Females of the latter two species (chimpanzees and orangutans) show also increased levels of cortisol metabolites when coerced, potentially reflecting elevated stress (Muller *et al*., 2007; Kunz *et al*., 2021a). These observations are reminiscent of the psychological and physiological consequences of sexual coercion for women (World Health Organization, 2012; Chéa *et al*., 2023; Tarzia & McKenzie, 2024).

## IV. FEMALE COUNTER‐STRATEGIES TO SEXUAL COERCION

Sexual conflict is usually unresolved (Chapman *et al*., 2003), since the evolutionary advantages gained by one sex over the other are unlikely to lead to a cessation of antagonistic co‐evolution. Accordingly, females able to express mate preference, alleviating the costs of sexual coercion, should have a selective advantage. In fact, females can commonly express mate preference even amid sexual coercion (Bisazza *et al*., 2001). For example, the ornaments of sexually coercive male guppies (*Poecilia reticulata*), mallards (*Anas platyrhynchos*) and mandrills are strikingly brighter than those of females, and previous work has suggested that they have likely been the target of female mate choice (Endler, 1980; Omland, 1996; Setchell, 2005; Smit, 2022, 2024). This observation is in line with the coercion‐avoidance hypothesis which posits that females should use male viability indicators (such as ornaments) as criteria for male quality, because in contrast to male body size and armaments, male viablty indicators cannot facilitate male sexual coercion of females (Pradhan & van Schaik, 2009).

**Fig. 2:**
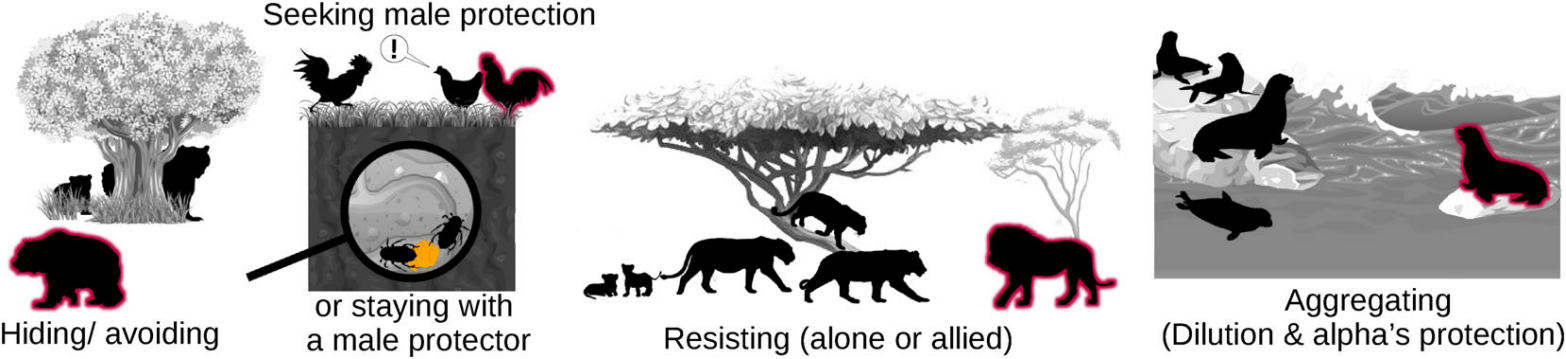
Common female counter‐strategies to sexual coercion. In each panel, coercive males are highlighted with red colour and look to the left.

Females are expected to develop physiological adaptations and behavioural counter‐strategies to sexual coercion (Muller *et al*., 2009b; Wrangham & Muller, 2009) that can increase their reproductive control and facilitate female mate choice. The various counter‐strategies against infanticide have been reviewed previously (Agrell *et al*., 1998; Ebensperger, 1998; Palombit, 2012, 2015) but reports of counter‐strategies against other sexually coercive strategies can be found mostly in taxon‐specific literature (e.g. primates; Muller & Wrangham, 2009). An increasing number of studies show that similar female counter‐strategies evolve against different male sexually coercive strategies across taxa (Fig. 2).

### (1) Deception: promiscuity, timing and pseudo‐oestrus

Females can mate promiscuously to appease and/or deceive coercive males (van Schaik & Janson, 2000; Wolff & Macdonald, 2004; Watters, 2005; Clarke *et al*., 2009). Female promiscuous mating is observed in carnivores (Dahle & Swenson, 2003; Dugdale, Griffiths & Macdonald, 2011), rodents (Hoogland, Wolff & Sherman, 2007), primates (Soltis *et al*., 2000), cetaceans (Connor *et al*., 1996), and other taxa (Stacey, 1982; Wolff & Macdonald, 2004; Fitzpatrick *et al*., 2009). Its efficiency as a counter‐strategy to coercion might depend on various ecological, demographic or morphological factors. It may not be an efficient counter‐strategy if the cost of multiple matings is greater than the cost of avoidance or resistance to coercive males [Rowe, 1992; see also ‘convenience polyandry’ hypothesis (Huchard *et al*., 2012)]. Additionally, if females are not able to mate with all or most potentially coercive males due to male density and movement patterns, they may develop alternative counter‐strategies (Harcourt & Greenberg, 2001). Along with promiscuous mating, females in some carnivores and rodents can produce litters sired by multiple males, confusing paternity and reducing the risk of infanticide (Bellemain, Swenson & Taberlet, 2006; Ishibashi & Saitoh, 2008; Thonhauser *et al*., 2013). Nevertheless, the production of multiple paternity broods might also reflect successful male harassment and female concession to mating with multiple males as a compromise (Lee & Hays, 2004; Sztatecsny *et al*., 2006).

At first glance, female promiscuity may seem detrimental for female mate choice as females seem to trade their reproductive control for decreased direct costs of coercion. Yet, females might be able to express mate preferences amid male coercion and promiscuous mating. Specifically, they can adapt the timing of mating with different males to bias conception probability towards preferred males while deceiving undesired ones. For example. they can use graded chemical (odours; Petrulis, 2013), visual (e.g. perineal sexual swellings; Barelli *et al*., 2007) or behavioural (Tiddi, Wheeler & Heistermann, 2015) signals of fertility that may allow them to manipulate male mating and coercive behaviour. If these signals reflect female fertility reliably, preferred, dominant, males can guard and mate with females when their signals peak, while the females can still mate with less‐desired males during non‐peak signal times, appeasing these males and reducing their coercive tendencies. For example, some female primates can use perineal sexual swellings as reliable graded signals of fertility and mate with subordinate males at non‐peak swelling times, offering to these males a non‐zero probability of paternity (Nunn, 1999) that might be sufficient to make them reluctant to commit infanticide.

Females can also deceive males and reduce coercion costs by blurring their peak fertility signals or suppressing such signals overall (see ‘concealed ovulation’ in mammals; Burley, 1979). After a male take‐over, lactating lionesses and female hamadryas baboons often cease their post‐partum amenorrhea and show heightened sexual activity that is not always associated with increased fertility and accelerated reproduction (Packer & Pusey, 1983; Zinner & Deschner, 2000) but allows females to deceive the new male and prevent infanticide (‘pseudo‐oestrus’; Fig. 1). Alternatively, females can wean their infants abruptly after a male take‐over (ursine colobus monkeys, *Colobus vellerosus*; Teichroeb & Sicotte, 2008), although this strategy may have detrimental consequences for infant survival. In some primates, females can exhibit post‐conception mating that deceives undesired males about their probability of paternity and reduces the risk of infanticide (Palombit, 2012, 2015). Female European badgers (*Meles meles*) can express mate preference, confuse paternity and reduce the risk of infanticide by conceiving during gestation and delaying implantation of their fertilised eggs (Yamaguchi, Dugdale & Macdonald, 2006).

Females can reduce the costs of other sexually coercive strategies than infanticide through similar counter‐strategies. Female chimpanzee resistance rates are lower in non‐periovulatory periods when conception is less likely (Stumpf & Boesch, 2005). Accordingly, the willingness of female Bornean orangutans to mate with non‐prime males is usually higher in non‐periovulatory periods (Knott *et al*., 2010), and in combination with concealed ovulation (Fox, 1998), it may allow female orangutans to bias fertilisation towards preferred males while appeasing coercive undesired males and reducing (the costs of) forced copulations.

Even when (promiscuous) females can not adapt the timing of mating with different males, they can express postcopulatory mate choice. Females in various frog species can release fewer eggs when amplexed by undesired and occasionally heterospecific coercive males (Reyer, Frei & Som, 1999; Roesli & Reyer, 2000; Hettyey & Pearman, 2003; Hettyey *et al*., 2009). Sexually coerced female feral fowl (*Gallus gallus domesticus*) and promiscuous female earwigs (*Euborellia plebeja*) can bias sperm retention in favour of preferred males (Pizzari & Birkhead, 2000). Women can secretly seek clinical support for contraception (Miller *et al*., 2007; Alvergne & Lummaa, 2010; Nikolajski *et al*., 2015) or intervention for abortions (Moore, Frohwirth & Miller, 2010). These female strategies can reduce the indirect costs of sexual coercion, offering increased female reproductive control. Additionally, they might reduce the benefits of sexual coercion for males which can reduce, in turn, the frequency of male coercive mating attempts and the associated direct costs to females. Finally, if females can efficiently express postcopulatory mate preference, they might not need to express precopulatory mate preference (Johannesson *et al*., 2016). Therefore, they might comply with male mating attempts, reducing any direct costs of active resistance to them.

### (2) Avoidance of undesired males

Given that the ultimate function of sexual coercion is the increase of male perpetrator fitness, females might use honest signals to inform males that they are not receptive in order to prevent ill‐fated coercive male mating attempts and reduce any direct costs of coercion. In the frog *Pelophylax nigromaculatus*, post‐spawning female vocalisations serve to deter males from approaching and attempt coercive amplexus (Itoh, 2023). Furthermore, females can physically avoid coercive males [insects (Rowe *et al*., 1994; McLain & Pratt, 1999)]. Evidence from humpback whales (Derville, Torres & Garrigue, 2018), lions (Dejeante *et al*., 2024), tigers (*Panthera tigris*; Singh *et al*., 2014), polar bears [*Ursus maritimus* (Stirling, Andriashek & Calvert, 1993; Clark & Stirling, 1998)], and brown bears (*Ursus arctos*; Penteriani *et al*., 2024) suggests that individual females can reduce the risk of infanticide by adapting their space use to avoid interactions with males. Female leaf beetles, *Leptinotarsa undecimlineata*, move to plants with fewer males and more females, likely to avoid male harassment (Muniz *et al*., 2018). Accordingly, infanticide in lions (Chakrabarti *et al*., 2021) and sexual harassment in guppies (Darden & Croft, 2008), bottlenose dolphins (Galezo, Krzyszczyk & Mann, 2018) and botos (*Inia geoffrensis*; Martin & da Silva, 2004) have likely promoted spatial segregation between females and males. Studies in more species have documented a relevant link between sexual coercion and temporal segregation of females and males (Wearmouth *et al*., 2012).

When females live in groups with males, sexual coercion can also influence female dispersal decisions (Smuts & Smuts, 1993). After male take‐overs, when the risk of infanticide is high, female primates without infants usually stay with the immigrant male but lactating or pregnant females can leave their groups with the ousted male (Winkler, Loch & Vogel, 1984; Jack & Fedigan, 2009; Zhao, Borries & Pan, 2011). Such female emigration not only increases the probability of survival of the fetuses or infants of emigrating females but it might also make new males cease their attacks on infants of any remaining females (Xiang *et al*., 2022); that is, males might prefer to delay reproduction waiting for females to wean their current infants, rather than to risk losing all (lactating) females to the ousted male. Male take‐overs might also instigate lactating females to leave their groups even without any males (Rudran, 1973; Packer & Pusey, 1983; Sterck & Korstjens, 2000) but this strategy may be too risky when male protection is crucial for female survival.

Fine‐grained observations in well‐studied species have revealed further male coercion avoidance strategies which might be common in other species but hard to document (Muller *et al*., 2009b). Female guppies seem to be able to reduce the costs of harassment through phenotypic plasticity and particularly by improving their swimming efficiency (i.e. using less oxygen to swim at a given speed) allowing them to avoid males (Killen *et al*., 2016). Women have likely evolved psychological mechanisms that promote behaviours that can decrease the risk of rape (Chavanne & Gallup, 1998; Bröder & Hohmann, 2003; Garver‐Apgar, Gangestad & Simpson, 2007), for example, by allowing them to assess the risk of sexual coercion under certain circumstances or by particular men (McKibbin *et al*., 2008). Other female primates might escape males by climbing trees. Female mandrills and chacma baboons who face male sexual intimidation (Baniel *et al*., 2017; Smit *et al*., 2022a), can take advantage of their lighter weight and smaller size (Setchell *et al*., 2001) to retreat to thin branches where males cannot reach them without risking a fall (N. Smit, personal observations; Kitchen *et al*., 2009). Finally, chimpanzee mothers with dependent infants decrease their association with males who improve their ranks (and are more likely to commit infanticide) and increase their association with potential male protectors (Lowe, Hobaiter & Newton‐Fisher, 2019).

Two recent studies on Iberian ribbed newts (*Pleurodeles walt*; Janssenswillen & Bossuyt, 2016) and European common frogs (Dittrich & Rödel, 2023) showed that females can use tonic immobility to escape male harassment (‘sexual death feigning’). Similar behaviours have been described in various species of dragonflies (Wildermuth *et al*., 2019). Although these examples are reminiscent of the death‐feigning of males in order to avoid sexual cannibalism in some spiders (Bilde *et al*., 2005; Hansen *et al*., 2008), the two mechanism are functionally distinct. In the first case, females feign death to avoid male sexual coercion. In the second case, males do not feign death to avoid female sexual coercion, since females do not try to mate with these males. Instead, female spiders may tend to avoid mating with these males and males use death feigning to deceive females and achieve mating (Bilde *et al*., 2005). Therefore, death feigning may be used both as a part of the male sexually coercive repertoire and as a female counter‐strategy to sexual coercion.

### (3) Association with male protectors

When females emigrate from groups to avoid coercion, they commonly choose to immigrate into groups that offer them better protection against male coercion (Stokes, 2004; Teichroeb, Wikberg & Sicotte, 2009; Robbins *et al*., 2009a; Yamagiwa, Kahekwa & Basabose, 2009; Pavé *et al*., 2012; Breuer, Robbins & Robbins, 2016). For example, in gorillas (genus *Gorilla*) where out‐group males constitute an infanticidal threat, females prefer immigrating into groups with more males (Robbins *et al*., 2009b) and actively compete for access to male protectors (Smit & Robbins, 2024). Female–male associations that protect females from coercion can, in turn, have a positive impact on female foraging efficiency (Wilcox, 1984), body condition (Linklater *et al*., 1999) and, ultimately, fitness. Males that help females protect their offspring against other infanticidal males are observed from burying beetles (*Nicrophorus pustulatus*; Trumbo, 2007) to several mammals (Hrdy, 1974; Watts, 1990; Borries *et al*., 1999; Bellemain *et al*., 2005; Swedell & Saunders, 2006; Palombit, 2009; Fruteau, Range & Noë, 2010; Fedigan & Jack, 2013). Females can reduce the risk of sexual harassment through long‐term associations with males in plains zebras (*Equus burchelli*) and feral horses (*Equus caballus*; Rubenstein, 1986, 1994) or short‐term associations with males in certain pinnipeds (Boeuf & Mesnick, 1991), Kafue lechwe (*Kobus leche kafuensis*; Nefdt, 1995) and female Sumatran orangutans (*Pongo pygmaeus abelii*; Fox, 2002).

Females can actively seek male protection. In feral fowls (Pizzari & Birkhead, 2000) and other birds and primates [Cox & Le Boeuf, 1977; see also Dittrich & Rödel (2023) for an example in amphibians], females utter distress calls when mounted by undesired, low‐ranking or young males, increasing the likelihood of dominant male interventions that reduce the likelihood of successful copulation with the undesired male. Indeed, male mallards often intervene aggressively in attempts of forced copulation of other males (Barash, 1977). Similarly, Asiatic lionesses roar more frequently when in oestrus, likely to solicit attention from several males (Chakrabarti, Banerjee & Jhala, 2023), incite male–male competition and indirectly ‘choose’ their mates and protectors.

### (4) Female aggregation: dilution, synchrony and female–female coalitions

The incitation of male–male competition and later mating with powerful males likely represents the expression of female mate preferences amid male sexual coercion (Cox & Le Boeuf, 1977; Kuester & Paul, 1992). Females might incite male–male competition by aggregating in space and thus attract multiple males to the same location. While the risk of sexual coercion can increase with male‐biased operational sex ratio (less receptive females per male; Golubović *et al*., 2018), female aggregation and the consequent spatial clustering of males can distract males from actively pursuing females, may promote the formation of male hierarchies and ultimately may provide female protection from dominant males against other, undesired, males (Pilastro *et al*., 2003). Based on this observation, previous studies have suggested that sexual harassment can create the social potential for the evolution of polygyny (Cassini, 2020).

Female grouping decisions can have further influence on sexual coercion. Females might aggregate in order to join forces and defend themselves (and their infants) against coercive males or simply dilute the risks and costs of sexual coercion (Connor, Read & Wrangham, 2000; Cords & Fuller, 2010). In poeciliid fish (Dadda, 2015), red deer (Carranza & Valencia, 1999) and several pinnipeds (Galimberti, Boitani & Marzetti, 2000a; Pistorius *et al*., 2001; McMahon & Bradshaw, 2004; Wolf, Kauermann & Trillmich, 2005; Cappozzo *et al*., 2008), individual females face less male harassment when they are in larger groups. The presence of other females can reduce male harassment and alleviate the associated reduction in foraging efficiency in mosquitofish (Gambusia holbrooki; Pilastro et al., (2003). Infants of female southern sea lions (*Otaria byronia*) breeding in large colonies are killed by males less often than those born in ‘solitary mating pairs’ (Campagna *et al*., 1992), but a general association between the risk of infanticide and female gregariousness has been previously questioned (Lukas & Huchard, 2014). Finally, females might also synchronise their oestrus (Boness *et al*., 1995; Clarke, Henzi & Barrett, 2012), so that ‘short‐term’ male sexual coercion, like forced copulation or sexual harassment, is diluted among several females. For example, female southern elephant seals (*Mirounga leonina*) synchronise their breeding activities and prefer breeding in large groups where encounters with subordinate males and sexual harassment are less likely (Galimberti *et al*., 2000a).

Certain studies in humans have found a positive correlation between the probability of sexual coercion and social isolation (Cense & Brackenridge, 2001). Contrarily, socially connected women can use ‘coalitions’ to retaliate against coercive men (Gruber & Bjorn, 1986; Smuts, 1992; Folke *et al*., 2020), reminiscent of female lionesses which have higher chances of repelling infanticidal males when living in larger prides (Grinnell & McComb, 1996). In line with these observations, in species with frequent female coalitions against males, such as bonobos (Tokuyama & Furuichi, 2016) and spotted hyaenas (Strauss & Holekamp, 2019), sexual coercion is often negligible (East *et al*., 2003; Paoli, 2009). Accordingly, when females live in groups with kin, with whom they can form coalitions, they may be better protected against coercive males (Smuts, 1992).

### (5) Active resistance

The most evident female counter‐strategy to male sexual coercion is a physical struggle against coercive males (Arnqvist, 1992; Wolff & Peterson, 1998; Lötter & Pillay, 2012; Oleinichenko, 2012), with (Morelli *et al*., 2009; Cords & Fuller, 2010) or without (Knott *et al*., 2010) the help of other females. In presence of males, females might maintain closer spatial proximity to their infants for protection (Scott *et al*., 2023). When females actively resist coercive attempts, their body size, dominance over males (Ichino, 2005) or their bonds with other females (Connor *et al*., 2000) might prove crucial. However, females can attempt to, and occasionally succeed in, defending themselves and their infants even from higher‐ranking or physically superior male coercers (Fruteau *et al*., 2010). During infant defence, even unrelated lionesses (Grinnell & McComb, 1996) or female primates (Fruteau *et al*., 2010; Perry, 2012) can form alliances against male coercers and in the latter they can be also joined by juveniles (Xiang *et al*., 2022). In feral fowl (Pizzari & Birkhead, 2000) and Bornean orangutans (Knott *et al*., 2010), females resist male forced copulation and occasionally manage to reduce copulation time. Notably, female resistance is not equally probable against all males (Fox, 1998). Extra‐pair copulation in birds is more often forced than within‐pair copulation (McKinney & Stolen, 1982; Sorenson, 1994; Adler, 2010) and female southern elephant seals protest more against coercive behaviours of lower‐ranking males (Galimberti *et al*., 2000a). These resisting patterns likely reflect female mate preferences and question the hypothesis that female resistance is a process of mate assessment (Knott & Kahlenberg, 2007; Knott, 2009; Muller *et al*., 2009b).

Certain morphological adaptations can help females resist male sexually coercive attempts (Drea & Wallen, 2003; Stumpf *et al*., 2011). First, females can develop anatomical traits that complicate mating without female cooperation. Female waterfowls have developed vaginal spiral‐like structures coiling in the opposite direction to the male phallus (Brennan *et al*., 2007). Female water striders (family Gerridae) have developed abdominal spines which help them to thwart harassing males (Arnqvist & Rowe, 1997). The famous ‘pseudo‐penis’ of female spotted hyenas prevents intromission without female cooperation (East, Hofer & Wickler, 1993). Second, females can develop thicker tissues that reduce the likelihood of injury from male sexual coercion, potentially reducing also the costs of female resistance. Female round stingrays (*Urolophus halleri*; Nordell, 1994) and blue sharks (*Prionace flauca*; Pratt Jr, 1979) have thicker skin that can protect them against male biting during mating. Similarly, females in species of seed beetles with more spiny male genitalia have a larger amount of connective tissue in their copulatory ducts (Rönn, Katvala & Arnqvist, 2007). The latter trait may increase female reproductive control as it can mitigate the costs of injury, allow females to mate with other males and decrease the ability of males to use their spiny genitalia as an anchor during mating. Finally, some female morphological traits may not assist female resistance to male coercion, but can be used to attract less male coercion in the first place. For example, male‐like females face less sexual harassment in some insects (Robertson, 1985; Cook *et al*., 1994).

### (6) Can mating with coercive males occasionally reflect female choice rather than concession?

Despite the great diversity of counter‐strategies to sexual coercion, females might be unable to avoid mating with coercive males or they might be able to avoid it only with prohibitively high costs. In these cases, mating with the most coercive males might not be additionally detrimental for female fitness, if females' sons inherit the highly sexually coercive phenotype that can boost their reproductive success (Fisher, 1915). A distinct, but relevant, mechanism might function when females are observed to mate with males who have previously killed their infants (Lukas & Huchard, 2014). Females might do so, not (only) because these males constitute the single or ‘least bad’ possible option, but also (or instead) because these males are more aggressive or powerful and might produce sons with high fitness potential. Therefore, females may mate with those among the coercive males who can at least offer them some fitness benefits (‘best of a bad job’).

Females might also indirectly choose to mate with coercive males by choosing other male traits linked with the coercive phenotype, such as male overall aggressiveness which may serve to protect females and their offspring efficiently against predators or other males. In this context, assuming an association between male sexual coercion rates and overall male aggressiveness (which is not always the case; Smit et al. 2022a, females might reinforce an evolutionary feedback loop that sustains sexual coercion by choosing aggressive males. Then, if the fitness benefits from choosing such males (e.g. producing successful, sexually coercive, sons) are greater than the costs of their mate choice restriction through male coercion, the functional component of coercion might be wiped out. Contrarily, if the costs are greater, sexual coercion may still act as a prominent evolutionary force. In a relevant observation, lionesses delay their reproduction with infanticidal males after take‐overs (Packer & Pusey, 1983), potentially to evaluate the protective potential of the new males and minimise the likelihood of further take‐overs and infanticides (see also Bellemain *et al*., 2005; Izar *et al*., 2009). If the ability of male lions to defend actively a pride against rival male intruders is correlated with their own ability to intrude and claim prides from rival males, then lionesses' mate choice might indirectly sustain the infanticidal male phenotype.

## V. INDIVIDUAL AND SPECIES‐LEVEL PREDICTORS OF SEXUAL COERCION

**Fig. 3:**
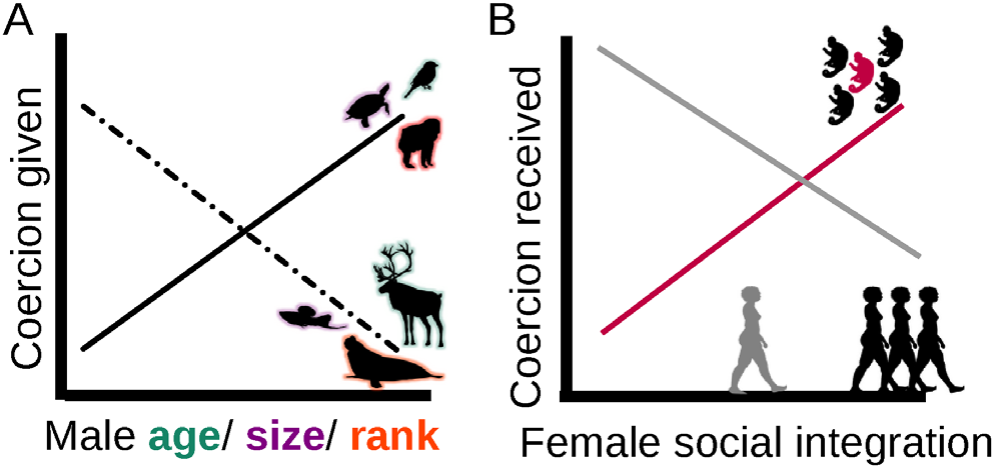
Traits that make (A) males more/less coercive and (B) females more/less coerced. In A, the ‘capacity coercion’ hypothesis is represented by the solid line (positive slope: older/bigger/more dominant males are more coercive) and the ‘opportunistic coercion’ hypothesis by the dashed line (negative slope: younger/smaller/less‐dominant males are more coercive). In B, females that receive more coercion are coloured according to the respective line: social integration is linked to more (red line; white‐faced capuchins; Kalbitzer *et al*., 2017) or less (grey line; humans; Cense & Brackenridge, 2001) received coercion.

Not all mating attempts or not all males are equally coercive, even in species where sexual coercion is widespread. Two contrasting hypothesised mechanisms may influence male coercive phenotypes (Fig. 3A). First, since sexual coercion is adaptive, males would be expected to be more coercive when their capacity or chance to exercise coercion is higher. Various examples support this ‘capacity coercion’ hypothesis. In chimpanzees, males use less affiliation and more aggression to obtain matings with females as they age and gain physical strength (Reddy *et al*., 2021; but see also Muller *et al*., 2024). Older males in many birds coerce females more effectively and likely more frequently than younger ones (Westneat & Stewart, 2003; Girndt *et al*., 2018). In painted turtles (*Chrysemys picta*), small males use more courtship and large males use more coercion to increase their mating success (Moldowan, Brooks & Litzgus, 2020b). Alpha‐male mandrills exhibit the highest rates of sexual intimidation despite the fact that they have priority of access to females in oestrus (Setchell *et al*., 2005; Smit *et al*., 2022a). In humans, while rapists are often assumed to be mostly sexually deprived men (Thornhill & Wilmsen Thornhill, 1983), empirical studies find little, if any, support for this assumption (Lalumière *et al*., 1996; Thompson, 2009). Instead, acquaintance rapists, who constitute the majority of perpetrators, often are relatively sexually active both in terms of sexual interactions and number of partners (Thornhill & Wilmsen Thornhill, 1983; Kanin, 1985; Lalumière & Quinsey, 1996; Lalumière & Lalumiere, 2005). In particular, intimate partner violence and rape are common (Basile, 2002), that is, men are sexually coercive with their partners despite the mating access they have to them.

Second, and in contrast to the previous hypothesis, males who otherwise have few opportunities of mating would be expected to be the most coercive. Under this ‘opportunistic coercion’ hypothesis, one would expect young rather than prime‐aged, small rather than large, low‐ranking rather than high‐ranking and peripheral/immigrant rather than territorial/resident males to be the most coercive (see also Clutton‐Brock & Parker, 1992). Indeed, in certain ungulates, primates and pinnipeds, young males exhibit higher rates of sexual harassment than older males (Loy & Loy, 1977; Boeuf & Mesnick, 1991; Holand *et al*., 2006; Apio *et al*., 2007). In Northern mountain swordtail fish (*Xiphophorus nezahualcoyotl*) smaller males are more coercive than larger ones (Morris, Rios‐Cardenas & Darrah, 2008). In feral goats (*Capra hircus*; Saunders *et al*., 2005), some pinnipeds (Cassini, 1999, 2000) and orangutans (Knott, 2009), sexual harassment is performed mostly by subordinate (satellite) males. Finally, in sexually coercive birds, extra‐pair copulation is more often forced than in‐pair copulation (McKinney & Stolen, 1982; Sorenson, 1994). Importantly, the two hypothesised mechanisms that determine male coercive phenotypes might function simultaneously or their function might depend on the different coercive strategies: less‐powerful males with few mating opportunities might use short‐term and/or direct sexual coercion opportunistically, while more powerful males might have the latitude to use long‐term and/or indirect sexual coercion to secure exclusive mating access to the female targets. Detailed studies in different taxa could help determine when one hypothesised mechanism has stronger predictive power than the other and when the two mechanisms function simultaneously.

The female traits that attract male sexual coercion are even less explored than the male sexually coercive phenotypes [but see Thompson (2009) for a review on human rape]. These female traits might coincide with those generally selected by male mate preferences [e.g. female size, mass, rank, age or fecundity (Sargent, Gross & Van Den Berghe, 1986; Bonduriansky, 2001; Preston *et al*., 2005; Girard‐Buttoz *et al*., 2014)], which may, in turn, depend on the mating system (Thompson, 2009), but relevant studies are scarce. Male chimpanzees direct higher levels of sexual intimidation to parous females (Muller *et al*., 2007) who have a greater probability of successful reproduction than nulliparous females. Accordingly, male mandrills prefer to mate‐guard high‐ranking and parous females (Setchell & Jean Wickings, 2006) and direct higher levels of sexual intimidation to higher‐ranking females (Smit *et al*., 2022a). Surprisingly, female social integration is positively correlated with a risk of sexual intimidation in mandrills (Smit *et al*., 2023) and a risk of infanticide in white‐faced capuchins (*Cebus capucinus imitator*; Kalbitzer *et al*., 2017). These correlations likely reflect that the central spatial positioning associated with increased social integration can increase the likelihood of interaction of females with other groupmates, including coercive males. In line with this hypothesis, female grey‐headed flying foxes (*Pteropus poliocephalus*) are harassed by males more in central locations (Dorrestein *et al*., 2024). In humans, relevant results are mixed; sexual coercion appears to be more common against socially isolated women (Cense & Brackenridge, 2001) and women in supervisory positions (Folke *et al*., 2020; but see Schlegel & Barry, 1986) rather than socially integrated women and women employees. Overall, female rank and social integration, which generally have a beneficial imprint on female life history, might occasionally attract sexual coercion but they may also predict a better resistance potential to it (Fig. 3B). Future work may clarify the risk factors of sexual coercion and quantify the trade‐offs that females face between the benefits of rank, social integration or bonds and the costs of sexual coercion.

Apart from our lack of knowledge on the tendencies or risk factors of sexual coercion at the individual level, we also lack a general predictive framework. Different strategies (Clutton‐Brock & Parker, 1995) and rates (Glover & Crowley, 2017) of sexual coercion appear associated with different social (and mating) systems. Forced copulation is commonly observed in monogamous species (Barash, 1977; Mineau, McKinney & Derrickson, 1983; Basile, 2002). Sexual harassment, which targets immediate mating opportunities, is probably more common in species with short‐term associations of females and males (Clutton‐Brock & Parker, 1995). By contrast, sexual intimidation is expected to be more common when females and males form long‐lasting relationships or at least long‐lasting spatiotemporal associations. Sexual punishment might also require long‐lasting intersexual relationships but also male control over the target's sexual activity. Finally, mate‐guarding might only require female–male associations during female receptive periods. Future comparative analyses need to test these associations and improve our understanding on the ‘social conditions’ that can foster or prevent the evolution of different sexually coercive strategies.

Various studies have argued that sexual coercion is, in fact, an important driver of social evolution. They have proposed links between the evolution of social monogamy and the risk of sexual punishment (Prosen *et al*., 2004), infanticide (Opie *et al*., 2013), mate‐guarding (Brotherton & Komers, 2003) or forced copulation (Gowaty & Buschhaus, 1998); the evolution of lek‐breeding in ungulates and birds and the risk of sexual harassment (Clutton‐Brock, Price & MacColl, 1992; Clutton‐Brock, Deutsch & Nefdt, 1993); or the evolution of polygyny and the risk of sexual harassment (Cassini, 2020). However, other studies have questioned these causal links (Bro‐Jørgensen, 2003; Lukas & Huchard, 2014). Our understanding of the ecology of sexual coercion will be drastically improved by studies exploring the presence and direction of any causal links between different sexually coercive strategies and mating or social systems.

## VI. CONCLUSIONS

(1) This review extends previous work providing compelling evidence of sexually antagonistic coevolution in the context of sexual coercion across animals (Smuts & Smuts, 1993; Clutton‐Brock & Parker, 1995; Arnqvist & Rowe, 2005; Muller & Wrangham, 2009; Cassini, 2021). Not all sexually coercive strategies involve extreme aggression levels, but they may still restrict female mate choice and offer reproductive advantages to male perpetrators. The majority of relevant empirical studies document mating rather than reproductive advantages for males and future work may address this gap of knowledge using paternity analysis to quantify the reproductive success of coercive and less‐coercive males.
(2) The costs of sexual coercion for females are manifold. Direct costs, such as injuries, are easier to document than indirect and fitness costs. Yet, recent work has started documenting substantial female fitness costs from male sexual coercion (Gómez‐Llano *et al*., 2024). Especially in slowly reproducing species where females produce a few offspring in their lifetime, coercion costs potentially act as a strong selective force on the evolution of female counter‐strategies. Future studies need to investigate female fitness costs from different male sexually coercive strategies, comparing the reproductive success of females that face sexual coercion with those that reproduce more freely.
(3) Similar female counter‐strategies, including well‐known female mating strategies which are often not recognised as adaptations against sexual coercion, can evolve against different male sexually coercive strategies. These counter‐strategies can serve different, but interrelated, purposes. They might act as a buffer to direct female costs, such as injuries, or to increase the costs/risks for males, making males reluctant to use coercion. Most importantly, they might increase the potential for female mate choice, reducing the functional power of sexual coercion. Altogether, the variety of counter‐strategies observed across taxa likely suggests detrimental consequences of coercion for females and the importance of female reproductive control for female fitness.
(4) The combination of different coercive strategies (e.g., chimpanzees: mate‐guarding, sequestering, sexual intimidation) or different counter‐strategies (e.g., lionesses: pseudo‐oestrus, inciting male–male competition, female–female coalitions) in some well‐studied species suggests that coercive males and coerced females can rely on multiple strategies to increase their reproductive control. In humans, we also observe an array of male coercive strategies including mate‐guarding, forced copulation, sexual punishment and other forms of sexual violence which resemble sexual harassment and intimidation (Buss, 2002; Muller & Wrangham, 2009; Barbaro & Shackelford, 2016). Accordingly, women can use contraception and seek social support against coercers and they have developed psychological mechanisms that might facilitate coercion avoidance.
(5) Beyond the study of the benefits of coercion for males and the costs for females in well‐studied species, future work may extend the investigation of sexual coercion to other taxa, such as arachnids, molluscs and crustaceans. The expansion of studies on sexual coercion may improve our understanding of the individual traits that make males more coercive and females more coerced. Finally, future work may develop a methodological framework for the study of a lack of sexual coercion. Studies that report a lack of sexual coercion will facilitate comparative analyses that can shed light on the causal relationships between different strategies of sexual coercion and the evolution of different social and mating systems.

## References

[brv70013-bib-0001] Adler, M. (2010). Sexual conflict in waterfowl: why do females resist extrapair copulations? Behavioral Ecology 21, 182–192.

[brv70013-bib-0002] Afton, A. D. (1985). Forced copulation as a reproductive strategy of male lesser scaup: a field test of some predictions. Behaviour 92, 146–167.

[brv70013-bib-0003] Agoramoorthy, G. , Mohnot, S. M. , Sommer, V. & Srivastava, A. (1988). Abortions in free ranging Hanuman langurs (*Presbytis entellus*) – a male induced strategy? Human Evolution 3, 297–308.

[brv70013-bib-0004] Agrell, J. , Wolff, J. O. & Ylönen, H. (1998). Counter‐strategies to infanticide in mammals: costs and consequences. Oikos 83, 507–517.

[brv70013-bib-0005] Ainsworth, S. E. & Maner, J. K. (2012). Sex begets violence: mating motives, social dominance, and physical aggression in men. Journal of Personality and Social Psychology 103, 819–829. 22823293 10.1037/a0029428

[brv70013-bib-0006] Alberts, S. C. , Altmann, J. & Wilson, M. (1996). Mate guarding constrains foraging activity of male baboons. Animal Behaviour 51, 1269–1277.

[brv70013-bib-0007] Alcock, J. (1994). Postinsemination associations between males and females in insects: the mate‐guarding hypothesis. Annual Review of Entomology 39, 1–21.

[brv70013-bib-0008] Alcock, J. & Forsyth, A. (1988). Post‐copulatory aggression toward their mates by males of the rove beetle *Leistotrophus versicolor* (Coleoptera: Staphylinidae). Behavioral Ecology and Sociobiology 22, 303–308.

[brv70013-bib-0009] Alvergne, A. & Lummaa, V. (2010). Does the contraceptive pill alter mate choice in humans? Trends in Ecology & Evolution 25, 171–179. 19818527 10.1016/j.tree.2009.08.003

[brv70013-bib-0010] Amann, A. L. , Pines, M. & Swedell, L. (2017). Contexts and consequences of takeovers in hamadryas baboons: female parity, reproductive state, and observational evidence of pregnancy loss. American Journal of Primatology 79, e22649. 10.1002/ajp.22649 28395395

[brv70013-bib-0011] Andelman, S. J. (1987). Evolution of concealed ovulation in vervet monkeys (*Cercopithecus aethiops*). The American Naturalist 129, 785–799.

[brv70013-bib-0012] Andersson, J. , Borg‐Karlson, A.‐K. & Wiklund, C. (2000). Sexual cooperation and conflict in butterflies: a male–transferred anti–aphrodisiac reduces harassment of recently mated females. Proceedings of the Royal Society of London. Series B: Biological Sciences 267, 1271–1275. 10.1098/rspb.2000.1138 PMC1690675 10972120

[brv70013-bib-0013] Apio, A. , Plath, M. , Tiedemann, R. & Wronski, T. (2007). Age‐dependent mating tactics in male bushbuck (*Tragelaphus scriptus*). Behaviour 144, 585–610.

[brv70013-bib-0014] Arnqvist, G. (1992). Pre‐copulatory fighting in a water strider: inter‐sexual conflict or mate assessment? Animal Behaviour 43, 559–567.

[brv70013-bib-0015] Arnqvist, G. & Nilsson, T. (2000). The evolution of polyandry: multiple mating and female fitness in insects. Animal Behaviour 60, 145–164. 10973716 10.1006/anbe.2000.1446

[brv70013-bib-0016] Arnqvist, G. & Rowe, L. (1997). Sexual conflict and arms races between the sexes: a morphological adaptation for control of mating in a female insect. Proceedings of the Royal Society of London. Series B: Biological Sciences 261, 123–127.

[brv70013-bib-0017] Arnqvist, G. & Rowe, L. (2005). Sexual conflict. Princeton University Press, Princeton.

[brv70013-bib-0018] Arseneau‐Robar, T. J. M. , Taucher, A. L. , Müller, E. , van Schaik, C. , Bshary, R. & Willems, E. P. (2016). Female monkeys use both the carrot and the stick to promote male participation in intergroup fights. Proceedings of the Royal Society B: Biological Sciences 283, 20161817. 10.1098/rspb.2016.1817 PMC5136586 27881752

[brv70013-bib-0019] Baker, R. R. (1972). Territorial behaviour of the nymphalid butterflies, *Aglais urticae* (L.) and *Inachis io* (L.). Journal of Animal Ecology 41, 453–469.

[brv70013-bib-0020] Baniel, A. , Cowlishaw, G. & Huchard, E. (2017). Male violence and sexual intimidation in a wild primate society. Current Biology 27, 2163–2168.e3. 28690113 10.1016/j.cub.2017.06.013

[brv70013-bib-0021] Baniel, A. , Webb, C. E. , Cowlishaw, G. & Huchard, E. (2021). The submissive pattern of postconflict affiliation in asymmetric relationships: a test in male and sexually coerced female baboons. Animal Behaviour 175, 87–97.

[brv70013-bib-0022] Barash, D. P. (1977). Sociobiology of rape in mallards (*Anas platyrhynchos*): responses of the mated male. Science 197, 788–789. 17790773 10.1126/science.197.4305.788

[brv70013-bib-0023] Barbaro, N. & Shackelford, T. K. (2016). Female‐directed violence as a form of sexual coercion in humans (*Homo sapiens*). Journal of Comparative Psychology 130, 321–327. 27732014 10.1037/com0000038

[brv70013-bib-0024] Barelli, C. , Heistermann, M. , Boesch, C. & Reichard, U. H. (2007). Sexual swellings in wild white‐handed gibbon females (*Hylobates lar*) indicate the probability of ovulation. Hormones and Behavior 51, 221–230. 17137580 10.1016/j.yhbeh.2006.10.008

[brv70013-bib-0025] Bartoš, L. , Bartošová, J. , Chaloupková, H. , Dušek, A. , Hradecká, L. & Svobodová, I. (2016). A sociobiological origin of pregnancy failure in domestic dogs. Scientific Reports 6, 22188. 26917034 10.1038/srep22188 PMC4768179

[brv70013-bib-0026] Bartoš, L. , Bartošová, J. , Pluháček, J. & Šindelářová, J. (2011). Promiscuous behaviour disrupts pregnancy block in domestic horse mares. Behavioral Ecology and Sociobiology 65, 1567–1572.

[brv70013-bib-0027] Basile, K. C. (2002). Prevalence of wife rape and other intimate partner sexual coercion in a nationally representative sample of women. Violence and Victims 17, 511–524. 12477095 10.1891/vivi.17.5.511.33717

[brv70013-bib-0028] Bateman, A. J. (1948). Intra‐sexual selection in *Drosophila* . Heredity 2, 349–368. 18103134 10.1038/hdy.1948.21

[brv70013-bib-0029] Baxter, C. M. , Barnett, R. & Dukas, R. (2015). Aggression, mate guarding and fitness in male fruit flies. Animal Behaviour 109, 235–241.

[brv70013-bib-0030] Bellemain, E. , Swenson, J. E. & Taberlet, P. (2006). Mating strategies in relation to sexually selected infanticide in a non‐social carnivore: the brown bear. Ethology 112, 238–246.

[brv70013-bib-0031] Bellemain, E. , Zedrosser, A. , Manel, S. , Waits, L. P. , Taberlet, P. & Swenson, J. E. (2005). The dilemma of female mate selection in the brown bear, a species with sexually selected infanticide. Proceedings of the Royal Society B: Biological Sciences 273, 283–291. 10.1098/rspb.2005.3331 PMC1560043 16543170

[brv70013-bib-0032] Berger, J. (1983). Induced abortion and social factors in wild horses. Nature 303, 59–61. 6682487 10.1038/303059a0

[brv70013-bib-0033] Berger, J. (1986). Wild Horses of the Great Basin: Social Competition and Population Size. University of Chicago Press, Chicago.

[brv70013-bib-0034] Bertram, B. C. R. (1975). Social factors influencing reproduction in wild lions. Journal of Zoology 177, 463–482.

[brv70013-bib-0035] Bilde, T. , Tuni, C. , Elsayed, R. , Pekár, S. & Toft, S. (2005). Death feigning in the face of sexual cannibalism. Biology Letters 2, 23–25. 10.1098/rsbl.2005.0392 PMC1617195 17148316

[brv70013-bib-0036] Birkhead, T. R. , Johnson, S. D. & Nettleship, D. N. (1985). Extra‐pair matings and mate guarding in the common murre *Uria aalge* . Animal Behaviour 33, 608–619.

[brv70013-bib-0037] Bisazza, A. (1993). Male competition, female mate choice and sexual size dimorphism in poeciliid fishes. Marine & Freshwater Behaviour & Physiology 23, 257–286.

[brv70013-bib-0038] Bisazza, A. , Vaccari, G. & Pilastro, A. (2001). Female mate choice in a mating system dominated by male sexual coercion. Behavioral Ecology 12, 59–64.

[brv70013-bib-0039] Boeuf, B. J. L. & Mesnick, S. (1991). Sexual behavior of male northern elephant seals: I. Lethal injuries to adult females. Behaviour 116, 143–162.

[brv70013-bib-0040] Bonduriansky, R. (2001). The evolution of male mate choice in insects: a synthesis of ideas and evidence. Biological Reviews 76, 305–339. 11569787 10.1017/s1464793101005693

[brv70013-bib-0041] Boness, D. J. , Bowen, W. D. & Iverson, S. J. (1995). Does male harassment of females contribute to reproductive synchrony in the grey seal by affecting maternal performance? Behavioral Ecology and Sociobiology 36, 1–10.

[brv70013-bib-0042] Borries, C. , Launhardt, K. , Epplen, C. , Epplen, J. T. & Winkler, P. (1999). Males as infant protectors in Hanuman langurs (*Presbytis entellus*) living in multimale groups – defence pattern, paternity and sexual behaviour. Behavioral Ecology and Sociobiology 46, 350–356.

[brv70013-bib-0043] Brennan, P. L. R. , Prum, R. O. , McCracken, K. G. , Sorenson, M. D. , Wilson, R. E. & Birkhead, T. R. (2007). Coevolution of male and female genital morphology in waterfowl. PLoS One 2, e418. 17476339 10.1371/journal.pone.0000418 PMC1855079

[brv70013-bib-0044] Brereton, A. R. (1995). Coercion‐defence hypothesis: the evolution of primate sociality. Folia Primatologica 64, 207–214.

[brv70013-bib-0045] Breuer, T. , Robbins, A. M. & Robbins, M. M. (2016). Sexual coercion and courtship by male western gorillas. Primates 57, 29–38. 26483073 10.1007/s10329-015-0496-9

[brv70013-bib-0047] Bro‐Jørgensen, J. (2003). No peace for estrous topi cows on leks. Behavioral Ecology 14, 521–525.

[brv70013-bib-0048] Bro‐Jørgensen, J. & Pangle, W. M. (2010). Male topi antelopes alarm snort deceptively to retain females for mating. The American Naturalist 176, E33–E39. 10.1086/653078 20477537

[brv70013-bib-0046] Bröder, A. & Hohmann, N. (2003). Variations in risk taking behavior over the menstrual cycle: an improved replication. Evolution and Human Behavior 24, 391–398.

[brv70013-bib-0049] Brotherton, P. N. M. & Komers, P. E. (2003). In Monogamy: Mating Strategies and Partnerships in Birds, Humans and Other Mammals (eds C. Boesch and U. H. Reichard ), pp. 42–58. Cambridge University Press, Cambridge.

[brv70013-bib-0050] Brownmiller, S. (1975). Against our Will: Men, Women, and Rape. Simon & Schuster, New York.

[brv70013-bib-0051] Bruce, H. M. (1959). An exteroceptive block to pregnancy in the mouse. Nature 184, 105. 13805128 10.1038/184105a0

[brv70013-bib-0052] Burke, N. W. & Holwell, G. I. (2021). Male coercion and female injury in a sexually cannibalistic mantis. Biology Letters 17, 20200811. 33465328 10.1098/rsbl.2020.0811 PMC7876600

[brv70013-bib-0053] Burley, N. (1979). The evolution of concealed ovulation. The American Naturalist 114, 835–858.

[brv70013-bib-0054] Buss, D. M. (2002). Human mate guarding. Neuroendocrinology Letters 23, 23–29. 12496732

[brv70013-bib-0055] Camilleri, J. A. & Quinsey, V. L. (2009). Testing the cuckoldry risk hypothesis of partner sexual coercion in community and forensic samples. Evolutionary Psychology 7, 164–178.

[brv70013-bib-0056] Campagna, C. , Bisioli, C. , Quintana, F. , Perez, F. & Vila, A. (1992). Group breeding in sea lions: pups survive better in colonies. Animal Behaviour 43, 541–548.

[brv70013-bib-0057] Campagna, C. , Le Boeuf, B. J. & Cappozzo, H. L. (1988). Group raids: a mating strategy of male southern sea lions. Behaviour 105, 224–249.

[brv70013-bib-0058] Cappozzo, H. L. , Túnez, J. I. & Cassini, M. H. (2008). Sexual harassment and female gregariousness in the south American sea lion, *Otaria flavescens* . Naturwissenschaften 95, 625–630. 18392796 10.1007/s00114-008-0363-2

[brv70013-bib-0059] Carranza, J. & Valencia, J. (1999). Red deer females collect on male clumps at mating areas. Behavioral Ecology 10, 525–532.

[brv70013-bib-0060] Cartwright, R. & Sullivan, M. (2009). Associations with multiple male groups increase the energy expenditure of humpback whale (*Megaptera novaeangliae*) female and calf pairs on the breeding grounds. Behaviour 146, 1573–1600.

[brv70013-bib-0061] Cassini, M. (2000). A model on female breeding dispersion and the reproductive systems of pinnipeds. Behavioural Processes 51, 93–99. 11074314 10.1016/s0376-6357(00)00121-2

[brv70013-bib-0062] Cassini, M. H. (1999). The evolution of reproductive systems in pinnipeds. Behavioral Ecology 10, 612–616.

[brv70013-bib-0063] Cassini, M. H. (2020). A mixed model of the evolution of polygyny and sexual size dimorphism in mammals. Mammal Review 50, 112–120.

[brv70013-bib-0064] Cassini, M. H. (2021). Sexual aggression in mammals. Mammal Review 51, 247–255.

[brv70013-bib-0065] Cense, M. & Brackenridge, C. (2001). Temporal and developmental risk factors for sexual harassment and abuse in sport. European Physical Education Review 7, 61–79.

[brv70013-bib-0068] Chakrabarti, S. & Jhala, Y. V. (2019). Battle of the sexes: a multi‐male mating strategy helps lionesses win the gender war of fitness. Behavioral Ecology 30, 1050–1061.

[brv70013-bib-0066] Chakrabarti, S. , Banerjee, K. & Jhala, Y. (2023). The role of food and mates in shaping asiatic lion societies. In Social Strategies of Carnivorous Mammalian Predators: Hunting and Surviving as Families, pp. 47–88. Springer International Publishing AG, Cham.

[brv70013-bib-0067] Chakrabarti, S. , Bump, J. K. , Jhala, Y. V. & Packer, C. (2021). Contrasting levels of social distancing between the sexes in lions. iScience 24(5), 102406. 10.1016/j.isci.2021.102406. 34013168 PMC8113998

[brv70013-bib-0069] Chapman, T. , Arnqvist, G. , Bangham, J. & Rowe, L. (2003). Sexual conflict. Trends in Ecology & Evolution 18, 41–47.

[brv70013-bib-0070] Chase, R. & Blanchard, K. C. (2006). The snail's love‐dart delivers mucus to increase paternity. Proceedings of the Royal Society B: Biological Sciences 273, 1471–1475. 10.1098/rspb.2006.3474 PMC1560308 16777740

[brv70013-bib-0071] Chavanne, T. J. & Gallup, G. G. (1998). Variation in risk taking behavior among female college students as a function of the menstrual cycle. Evolution and Human Behavior 19, 27–32.

[brv70013-bib-0072] Chéa, M. , Bourguignon, C. , Bouvier, S. , Nouvellon, E. , Laurent, J. , Perez‐Martin, A. , Mousty, E. , Ripart, S. , Nikolaeva, M. G. , Khizroeva, J. , Bitsadze, V. , Makatsariya, A. & Gris, J.‐C. (2023). Intimate partner violence as a risk factor for venous thromboembolism in women on combined oral contraceptives: an international matched case‐control study. European Journal of Internal Medicine 122, 47–53. 38135584 10.1016/j.ejim.2023.12.016

[brv70013-bib-0414] Chung, W.‐S. , Kurniawan, N. D. , Marshall, N. J. , & Cortesi, F. (2025). Blue‐lined octopus Hapalochlaena fasciata males envenomate females to facilitate copulation. Current Biology, 35, R169–R170. 10.1016/j.cub.2025.01.027. 40068607

[brv70013-bib-0073] Clark, D. A. & Stirling, I. (1998). Habitat preferences of polar bears in the Hudson Bay lowlands during late summer and fall. Ursus 10, 243–250.

[brv70013-bib-0075] Clarke, P. M. R. , Henzi, S. P. & Barrett, L. (2012). Estrous synchrony in a nonseasonal breeder: adaptive strategy or population process? Behavioral Ecology 23, 573–581.

[brv70013-bib-0074] Clarke, P. , Pradhan, G. , van Schaik, C. , Muller, M. N. & Wrangham, R. W. (2009). Intersexual conflict in primates: infanticide, paternity allocation, and the role of coercion. In Sexual Coercion in Primates and Humans (eds M. N. Muller and R. W. Wrangham ), pp. 42–77. Harvard University Press, Cambridge.

[brv70013-bib-0078] Clutton‐Brock, T. H. & Parker, G. A. (1992). Potential reproductive rates and the operation of sexual selection. The Quarterly Review of Biology 67, 437–456.

[brv70013-bib-0079] Clutton‐Brock, T. H. & Parker, G. A. (1995). Sexual coercion in animal societies. Animal Behaviour 49, 1345–1365.

[brv70013-bib-0076] Clutton‐Brock, T. H. , Deutsch, J. C. & Nefdt, R. J. C. (1993). The evolution of ungulate leks. Animal Behaviour 46, 1121–1138.

[brv70013-bib-0077] Clutton‐Brock, T. H. , Green, D. , Hiraiwa‐Hasegawa, M. & Albon, S. D. (1988). Passing the buck: resource defence, lek breeding and mate choice in fallow deer. Behavioral Ecology and Sociobiology 23, 281–296.

[brv70013-bib-0080] Clutton‐Brock, T. H. , Price, O. F. & MacColl, A. D. C. (1992). Mate retention, harassment, and the evolution of ungulate leks. Behavioral Ecology 3, 234–242.

[brv70013-bib-0084] Connor, R. C. & Vollmer, N. (2009). Sexual coercion in dolphin consortships: a comparison with chimpanzees. In Sexual Coercion in Primates and Humans (eds M. N. Muller and R. W. Wrangham ), pp. 218–243. Harvard University Press, Cambridge.

[brv70013-bib-0082] Connor, R. C. , Richards, A. F. , Smolker, R. A. & Mann, J. (1996). Patterns of female attractiveness in Indian Ocean bottlenose dolphins. Behaviour 133, 37–69.

[brv70013-bib-0083] Connor, R. C. , Smolker, R. A. & Richards, A. F. (1992). Two levels of alliance formation among male bottlenose dolphins (*Tursiops* sp.). Proceedings of the National Academy of Sciences 89, 987–990. 10.1073/pnas.89.3.987 PMC48370 11607275

[brv70013-bib-0081] Connor, R. , Read, A. & Wrangham, R. (2000). Male reproductive strategies and social bonds. In Cetacean Societies: Field Studies of Dolphins and Whales, pp. 247–269. University of Chicago Press, Chicago.

[brv70013-bib-0085] Cook, S. E. , Vernon, J. G. , Bateson, M. & Guilford, T. (1994). Mate choice in the polymorphic African swallowtail butterfly, *Papilio dardanus*: male‐like females may avoid sexual harassment. Animal Behaviour 47, 389–397.

[brv70013-bib-0086] Cooper, M. A. , Aureli, F. & Singh, M. (2004). Between‐group encounters among bonnet macaques (*Macaca radiata*). Behavioral Ecology and Sociobiology 56, 217–227.

[brv70013-bib-0087] Cordero, A. (1999). Forced copulations and female contact guarding at a high male density in a calopterygid damselfly. Journal of Insect Behavior 12, 27–37.

[brv70013-bib-0088] Cordero, A. & Andrés, J. A. (2002). Male coercion and convenience polyandry in a calopterygid damselfly. Journal of Insect Science 2, 14. 15455048 10.1093/jis/2.1.14 PMC355914

[brv70013-bib-0089] Cords, M. & Fuller, J. L. (2010). Infanticide in Cercopithecus mitis stuhlmanni in the Kakamega Forest, Kenya: variation in the occurrence of an adaptive behavior. International Journal of Primatology 31, 409–431.

[brv70013-bib-0090] Cothran, R. (2020). Sexual selection and sexual conflict in crustaceans. In Reproductive Biology: The Natural History of the Crustacea (Volume 6, eds R. Cothran and M. Thiel ), pp. 305–331. Oxford University Press, Oxford.

[brv70013-bib-0091] Cox, C. R. & Le Boeuf, B. J. (1977). Female incitation of male competition: a mechanism in sexual selection. The American Naturalist 111, 317–335.

[brv70013-bib-0092] Crook, J. R. & Shields, W. M. (1985). Sexually selected infanticide by adult male barn swallows. Animal Behaviour 33, 754–761.

[brv70013-bib-0093] Dadda, M. (2015). Female social response to male sexual harassment in poeciliid fish: a comparison of six species. Frontiers in Psychology 6, 1453. 10.3389/fpsyg.2015.01453. 26483719 PMC4586586

[brv70013-bib-0094] Dahle, B. & Swenson, J. E. (2003). Seasonal range size in relation to reproductive strategies in brown bears *Ursus arctos* . Journal of Animal Ecology 72, 660–667. 30893970 10.1046/j.1365-2656.2003.00737.x

[brv70013-bib-0095] Daly, M. & Wilson, M. (1988). Homicide: Foundations of Human Behavior. Aldine de Gruyter, New York.

[brv70013-bib-0096] Darden, S. K. & Croft, D. P. (2008). Male harassment drives females to alter habitat use and leads to segregation of the sexes. Biology Letters 4, 449–451. 18682356 10.1098/rsbl.2008.0308 PMC2610095

[brv70013-bib-0097] Darden, S. K. , James, R. , Ramnarine, I. W. & Croft, D. P. (2009). Social implications of the battle of the sexes: sexual harassment disrupts female sociality and social recognition. Proceedings of the Royal Society B: Biological Sciences 276, 2651–2656. 10.1098/rspb.2009.0087 PMC2686652 19386653

[brv70013-bib-0098] Davidian, E. , Surbeck, M. , Lukas, D. , Kappeler, P. M. & Huchard, E. (2022). The eco‐evolutionary landscape of power relationships between males and females. Trends in Ecology & Evolution 37, 706–718. 35597702 10.1016/j.tree.2022.04.004

[brv70013-bib-0099] Davies, N. B. & Halliday, T. R. (1979). Competitive mate searching in male common toads, *Bufo bufo* . Animal Behaviour 27, 1253–1267.

[brv70013-bib-0100] Dean, M. D. (2013). Genetic disruption of the copulatory plug in mice leads to severely reduced fertility. PLoS Genetics 9, e1003185. 23341775 10.1371/journal.pgen.1003185 PMC3547826

[brv70013-bib-0101] DeGue, S. & DiLillo, D. (2005). ‘You would if you loved me’: toward an improved conceptual and etiological understanding of nonphysical male sexual coercion. Aggression and Violent Behavior 10, 513–532.

[brv70013-bib-0102] Dejeante, R. , Loveridge, A. J. , Macdonald, D. W. , Madhlamoto, D. , Valeix, M. & Chamaillé‐Jammes, S. (2024). Counter‐strategies to infanticide: the importance of cubs in determining lion habitat selection and social interactions. Journal of Animal Ecology 93, 159–170. 38174381 10.1111/1365-2656.14045

[brv70013-bib-0103] den Hollander, M. & Gwynne, D. T. (2009). Female fitness consequences of male harassment and copulation in seed beetles, *Callosobruchus maculatus* . Animal Behaviour 78, 1061–1070.

[brv70013-bib-0104] Derville, S. , Torres, L. G. & Garrigue, C. (2018). Social segregation of humpback whales in contrasted coastal and oceanic breeding habitats. Journal of Mammalogy 99, 41–54.

[brv70013-bib-0105] Dittrich, C. & Rödel, M.‐O. (2023). Drop dead! Female mate avoidance in an explosively breeding frog. Royal Society Open Science 10, 230742. 37830023 10.1098/rsos.230742 PMC10565404

[brv70013-bib-0106] Dixson, A. F. & Anderson, M. J. (2002). Sexual selection, seminal coagulation and copulatory plug formation in primates. Folia Primatologica 73, 63–69. 10.1159/000064784 12207054

[brv70013-bib-0107] Dorrestein, A. , Westcott, D. , Martin, J. M. , Phalen, D. , Rose, K. & Welbergen, J. A. (2024). Bat mating systems—a review and recategorisation. Ecology and Evolution 14, e70149. 39157663 10.1002/ece3.70149 PMC11327276

[brv70013-bib-0108] Drea, C. M. & Wallen, K. (2003). Female sexuality and the myth of male control. In Evolution, Gender, and Rape (ed. C. B. Travis ), pp. 29–60. MIT Press, Cambridge.

[brv70013-bib-0109] Dugdale, H. L. , Griffiths, A. & Macdonald, D. W. (2011). Polygynandrous and repeated mounting behaviour in European badgers, *Meles meles* . Animal Behaviour 82, 1287–1297.

[brv70013-bib-0110] Dukas, R. & Jongsma, K. (2012). Costs to females and benefits to males from forced copulations in fruit flies. Animal Behaviour 84, 1177–1182.

[brv70013-bib-0111] East, M. L. , Burke, T. , Wilhelm, K. , Greig, C. & Hofer, H. (2003). Sexual conflicts in spotted hyenas: male and female mating tactics and their reproductive outcome with respect to age, social status and tenure. Proceedings of the Royal Society of London. Series B: Biological Sciences 270, 1247–1254. 10.1098/rspb.2003.2363 PMC1691369 12816637

[brv70013-bib-0112] East, M. L. , Hofer, H. & Wickler, W. (1993). The erect ‘penis’ is a flag of submission in a female‐dominated society: greetings in Serengeti spotted hyenas. Behavioral Ecology and Sociobiology 33, 355–370.

[brv70013-bib-0113] Ebensperger, L. A. (1998). Strategies and counterstrategies to infanticide in mammals. Biological Reviews of the Cambridge Philosophical Society 73, 321–346.

[brv70013-bib-0114] Eberhard, W. G. (1985). Sexual Selection and Animal Genitalia. Harvard University Press, Cambridge, MA.

[brv70013-bib-0115] Emlen, S. T. & Wrege, P. H. (1986). Forced copulations and intra‐specific parasitism: two costs of social living in the white‐fronted bee‐eater. Ethology 71, 2–29.

[brv70013-bib-0116] Endler, J. A. (1980). Natural selection on color patterns in *Poecilia reticulata* . Evolution 34, 76–91. 28563214 10.1111/j.1558-5646.1980.tb04790.x

[brv70013-bib-0117] Englander, E. (2015). Coerced sexting and revenge porn among teens. In Bullying, Teen Aggression & Social Media (Volume 1), pp. 19–21. Civic Research Institute, Kingston, New Jersey.

[brv70013-bib-0118] Enigk, D. K. , Emery Thompson, M. , Machanda, Z. P. , Wrangham, R. W. & Muller, M. N. (2021). Female‐directed aggression by adolescent male chimpanzees primarily constitutes dominance striving, not sexual coercion. American Journal of Physical Anthropology 176, 66–79. 33938563 10.1002/ajpa.24296 PMC8376763

[brv70013-bib-0119] Farr, J. (1989). Sexual selection and secondary sexual differentiation in poeciliids: determinants of male mating success and the evolution of female choice. In Ecology and Evolution of Livebearing Fishes (Poeciliidae) (eds G. K. Meffe and F. F. Snelson ), pp. 91–123. Prentice Hall, Englewood Cliffs, NJ.

[brv70013-bib-0120] Fedigan, L. M. & Jack, K. M. (2013). Sexual conflict in white‐faced capuchins. In Evolution's Empress: Darwinian Perspectives on the Nature of Women (eds M. L. Fisher , J. R. Garcia and R. S. Chang ), pp. 281–303. Oxford University Press, New York.

[brv70013-bib-0121] Feh, C. & Munkhtuya, B. (2008). Male infanticide and paternity analyses in a socially natural herd of Przewalski's horses: sexual selection? Behavioural Processes 78, 335–339. 18328636 10.1016/j.beproc.2007.12.009

[brv70013-bib-0122] Feldblum, J. T. , Wroblewski, E. E. , Rudicell, R. S. , Hahn, B. H. , Paiva, T. , Cetinkaya‐Rundel, M. , Pusey, A. E. & Gilby, I. C. (2014). Sexually coercive male chimpanzees sire more offspring. Current Biology 24, 2855–2860. 25454788 10.1016/j.cub.2014.10.039 PMC4905588

[brv70013-bib-0123] Fisher, R. A. (1915). The evolution of sexual preference. The Eugenics Review 7, 184–192. 21259607 PMC2987134

[brv70013-bib-0124] Fitzpatrick, J. L. , Montgomerie, R. , Desjardins, J. K. , Stiver, K. A. , Kolm, N. & Balshines, S. (2009). Female promiscuity promotes the evolution of faster sperm in cichlid fishes. Proceedings of the National Academy of Sciences 106, 1128–1132. 10.1073/pnas.0809990106 PMC2633556 19164576

[brv70013-bib-0125] Foitzik, S. , Heinze, J. , Oberstadt, B. & Herbers, J. M. (2002). Mate guarding and alternative reproductive tactics in the ant *Hypoponera opacior* . Animal Behaviour 63, 597–604.

[brv70013-bib-0126] Folke, O. , Rickne, J. , Tanaka, S. & Tateishi, Y. (2020). Sexual harassment of women leaders. Daedalus 149, 180–197.

[brv70013-bib-0127] Fox, E. (2002). Female tactics to reduce sexual harassment in the Sumatran orangutan (*Pongo pygmaeus abelii*). Behavioral Ecology and Sociobiology 52, 93–101.

[brv70013-bib-0128] Fox, E. A. (1998). The Function of Female Mate Choice in the Sumatran Orangutan (Pongo Pygmaeus Abelii). Duke University, Durham, North Carolina.

[brv70013-bib-0129] Freed, L. A. (1986). Territory takeover and sexually selected infanticide in tropical house wrens. Behavioral Ecology and Sociobiology 19, 197–206.

[brv70013-bib-0130] Frère, C. H. , Krützen, M. , Kopps, A. M. , Ward, P. , Mann, J. & Sherwin, W. B. (2010). Inbreeding tolerance and fitness costs in wild bottlenose dolphins. Proceedings of the Royal Society B: Biological Sciences 277, 2667–2673. 10.1098/rspb.2010.0039 PMC2982034 20392729

[brv70013-bib-0131] Fruteau, C. , Range, F. & Noë, R. (2010). Infanticide risk and infant defence in multi‐male free‐ranging sooty mangabeys, *Cercocebus atys* . Behavioural Processes 83, 113–118. 19914358 10.1016/j.beproc.2009.11.004

[brv70013-bib-0132] Galezo, A. A. , Krzyszczyk, E. & Mann, J. (2018). Sexual segregation in indo‐Pacific bottlenose dolphins is driven by female avoidance of males. Behavioral Ecology 29, 377–386.

[brv70013-bib-0133] Galimberti, F. , Boitani, L. & Marzetti, I. (2000 *a*). Female strategies of harassment reduction in southern elephant seals. Ethology Ecology & Evolution 12, 367–388.

[brv70013-bib-0134] Galimberti, F. , Boitani, L. & Marzetti, I. (2000 *b*). The frequency and costs of harassment in southern elephant seals. Ethology Ecology & Evolution 12, 345–365.

[brv70013-bib-0136] Garver‐Apgar, C. E. , Gangestad, S. W. & Simpson, J. A. (2007). Women's perceptions of men's sexual coerciveness change across the menstrual cycle. Acta Psychologica Sinica 39, 536–540.

[brv70013-bib-0137] Gay, L. , Eady, P. E. , Vasudev, R. , Hosken, D. J. & Tregenza, T. (2009). Costly sexual harassment in a beetle. Physiological Entomology 34, 86–92.

[brv70013-bib-0138] Gibbon, C. D. F. (1997). The adaptive significance of monogamy in the golden‐reumped elephant‐shrew. Journal of Zoology 242, 167–177.

[brv70013-bib-0139] Girard‐Buttoz, C. , Heistermann, M. , Rahmi, E. , Agil, M. , Fauzan, P. A. & Engelhardt, A. (2014). Costs of and investment in mate‐guarding in wild long‐tailed macaques (*Macaca fascicularis*): influences of female characteristics and male–female social bonds. International Journal of Primatology 35, 701–724. 10.1007/s10764-014-9775-3 PMC4129240 25152554

[brv70013-bib-0140] Girndt, A. , Chng, C. W. T. , Burke, T. & Schroeder, J. (2018). Male age is associated with extra‐pair paternity, but not with extra‐pair mating behaviour. Scientific Reports 8, 8378. 29849085 10.1038/s41598-018-26649-1 PMC5976671

[brv70013-bib-0141] Glover, K. M. & Crowley, P. H. (2017). Female mate choice and the emergence of male coercion. Behavioral Ecology and Sociobiology 71, 181.

[brv70013-bib-0142] Goetz, A. T. & Shackelford, T. K. (2006). Sexual coercion and forced in‐pair copulation as sperm competition tactics in humans. Human Nature 17, 265–282. 26181473 10.1007/s12110-006-1009-8

[brv70013-bib-0143] Goetz, A. T. , Shackelford, T. K. , Romero, G. A. , Kaighobadi, F. & Miner, E. J. (2008). Punishment, proprietariness, and paternity: men's violence against women from an evolutionary perspective. Aggression and Violent Behavior 13, 481–489.

[brv70013-bib-0144] Gogliath, M. , Ribeiro, L. B. & Freire, E. M. X. (2010). Forced copulation attempt in the Blue‐tailed Lizard, *Micrablepharus maximiliani* (Reinhardt & Luetken, 1862) (Squamata, Gymnophthalmidae) in the Caatinga of Northeastern Brazil. Biota Neotropica 10, 347–350.

[brv70013-bib-0145] Golubović, A. , Arsovski, D. , Tomović, L. & Bonnet, X. (2018). Is sexual brutality maladaptive under high population density? Biological Journal of the Linnean Society 124, 394–402.

[brv70013-bib-0146] Gómez‐Llano, M. , Faria, G. S. , García‐Roa, R. , Noble, D. W. A. & Carazo, P. (2024). Male harm suppresses female fitness, affecting the dynamics of adaptation and evolutionary rescue. Evolution Letters 8, 149–160. 38370549 10.1093/evlett/qrac002 PMC10871930

[brv70013-bib-0147] Gowaty, P. A. & Buschhaus, N. (1998). Ultimate causation of aggressive and forced copulation in birds: female resistance, the CODE hypothesis, and social monogamy. American Zoologist 38, 207–225.

[brv70013-bib-0148] Gray, M. E. (2008). An infanticide attempt by a free‐roaming feral stallion (*Equus caballus*). Biology Letters 5, 23–25. 10.1098/rsbl.2008.0571 PMC2657763 19019779

[brv70013-bib-0149] Grinnell, J. & McComb, K. (1996). Maternal grouping as a defense against infanticide by males: evidence from field playback experiments on African lions. Behavioral Ecology 7, 55–59.

[brv70013-bib-0150] Gruber, J. E. & Bjorn, L. (1986). Women's responses to sexual harassment: an analysis of sociocultural, organizational and personal resource models. Social Science Quarterly 67, 814–826.

[brv70013-bib-0151] Hackländer, K. & Arnold, W. (1999). Male‐caused failure of female reproduction and its adaptive value in alpine marmots (*Marmota marmota*). Behavioral Ecology 10, 592–597.

[brv70013-bib-0152] Hansen, L. S. , Gonzales, S. F. , Toft, S. & Bilde, T. (2008). Thanatosis as an adaptive male mating strategy in the nuptial gift–giving spider *Pisaura mirabilis* . Behavioral Ecology 19, 546–551.

[brv70013-bib-0153] Harcourt, A. H. & Greenberg, J. (2001). Do gorilla females join males to avoid infanticide? A quantitative model. Animal Behaviour 62, 905–915.

[brv70013-bib-0154] Hennekam, S. & Bennett, D. (2017). Sexual harassment in the creative industries: tolerance, culture and the need for change. Gender, Work and Organization 24, 417–434.

[brv70013-bib-0155] Herberstein, M. E. , Wignall, A. E. , Nessler, S. H. , Harmer, A. M. T. & Schneider, J. M. (2012). How effective and persistent are fragments of male genitalia as mating plugs? Behavioral Ecology 23, 1140–1145.

[brv70013-bib-0157] Hettyey, A. & Pearman, P. B. (2003). Social environment and reproductive interference affect reproductive success in the frog *Rana latastei* . Behavioral Ecology 14, 294–300.

[brv70013-bib-0156] Hettyey, A. , Baksay, S. , Vági, B. & Hoi, H. (2009). Counterstrategies by female frogs to sexual coercion by heterospecifics. Animal Behaviour 78, 1365–1372.

[brv70013-bib-0158] Hiruki, L. M. , Stirling, I. , Gilmartin, W. G. , Johanos, T. C. & Becker, B. L. (1993). Significance of wounding to female reproductive success in Hawaiian monk seals (*Monachus schauinslandi*) at Laysan Island. Canadian Journal of Zoology 71, 469–474.

[brv70013-bib-0159] Holand, Ø. , Weladji, R. B. , Røed, K. H. , Gjøstein, H. , Kumpula, J. , Gaillard, J.‐M. , Smith, M. E. & Nieminen, M. (2006). Male age structure influences females' mass change during rut in a polygynous ungulate: the reindeer (*Rangifer tarandus*). Behavioral Ecology and Sociobiology 59, 682–688.

[brv70013-bib-0160] Holland, B. & Rice, W. R. (1998). Perspective: chase‐away sexual selection: antagonistic seduction versus resistance. Evolution 52, 1–7. 28568154 10.1111/j.1558-5646.1998.tb05132.x

[brv70013-bib-0161] Hoogland, J. L. , Wolff, J. & Sherman, P. (2007). Alarm calling, multiple mating, and infanticide among black‐tailed, Gunnison's, and Utah prairie dogs. In Rodent Societies: An Ecological and Evolutionary Perspective (eds J. O. Wolff and P. W. Sherman ), pp. 438–450. University of Chicago Press, Chicago.

[brv70013-bib-0162] Hooper, R. , Maher, K. , Moore, K. , McIvor, G. , Hosken, D. & Thornton, A. (2024). Ultimate drivers of forced extra‐pair copulations in birds lacking a penis: jackdaws as a case‐study. Royal Society Open Science 11, 231226. 38545615 10.1098/rsos.231226 PMC10966391

[brv70013-bib-0163] Hrdy, S. B. (1974). Male‐male competition and infanticide among the langurs (*Presbytis entellus*) of Abu, Rajasthan. Folia Primatologica 22, 19–58. 10.1159/000155616 4215710

[brv70013-bib-0164] Hrdy, S. B. (1979). Infanticide among animals: a review, classification, and examination of the implications for the reproductive strategies of females. Ethology and Sociobiology 1, 13–40.

[brv70013-bib-0165] Huchard, E. , Canale, C. I. , Le Gros, C. , Perret, M. , Henry, P.‐Y. & Kappeler, P. M. (2012). Convenience polyandry or convenience polygyny? Costly sex under female control in a promiscuous primate. Proceedings of the Royal Society B: Biological Sciences 279, 1371–1379. 10.1098/rspb.2011.1326 PMC3282357 21976684

[brv70013-bib-0166] Ichikawa, N. (1990). Egg mass destroying behaviour of the female giant water bug *Lethocerus deyrollei* Vuillefroy (*Heteroptera: Belostomatidae*). Journal of Ethology 8, 5–11.

[brv70013-bib-0167] Ichino, S. (2005). Attacks on a wild infant ring‐tailed lemur (*Lemur catta*) by immigrant males at Berenty, Madagascar: interpreting infanticide by males. American Journal of Primatology 67, 267–272. 16229008 10.1002/ajp.20183

[brv70013-bib-0168] Ishibashi, Y. & Saitoh, T. (2008). Effect of local density of males on the occurrence of multimale mating in gray‐sided voles (*Myodes rufocanus*). Journal of Mammalogy 89, 388–397.

[brv70013-bib-0169] Isvaran, K. (2005). Female grouping best predicts lekking in blackbuck (*Antilope cervicapra*). Behavioral Ecology and Sociobiology 57, 283–294.

[brv70013-bib-0170] Itoh, M. M. (2023). Female pond frog vocalisation deters sexual coercion by males. Behavioural Processes 210, 104905. 37301239 10.1016/j.beproc.2023.104905

[brv70013-bib-0171] Izar, P. , Stone, A. , Carnegie, S. & Nakai, É. S. (2009). Sexual selection, female choice and mating systems. In South American Primates: Comparative Perspectives in the Study of Behavior, Ecology, and Conservation (eds P. A. Garber , A. Estrada , J. C. Bicca‐Marques , E. W. Heymann and K. B. Strier ), pp. 157–189. Springer, New York.

[brv70013-bib-0172] Jack, K. M. & Fedigan, L. M. (2009). Female dispersal in a female‐philopatric species, *Cebus capucinus* . Behaviour 146, 471–497.

[brv70013-bib-0173] Jaeger, R. G. , Gillette, J. R. & Cooper, R. C. (2002). Sexual coercion in a territorial salamander: males punish socially polyandrous female partners. Animal Behaviour 63, 871–877.

[brv70013-bib-0174] Janssenswillen, S. & Bossuyt, F. (2016). Male courtship pheromones induce cloacal gaping in female newts (*Salamandridae*). PLoS One 11, e0144985. 26771882 10.1371/journal.pone.0144985 PMC4714853

[brv70013-bib-0175] Jindal, S. , Bose, A. P. H. , O'Connor, C. M. & Balshines, S. (2017). A test of male infanticide as a reproductive tactic in a cichlid fish. Royal Society Open Science 4, 160891. 28405376 10.1098/rsos.160891 PMC5383833

[brv70013-bib-0176] Johannesson, K. , Saltin, S. H. , Charrier, G. , Ring, A.‐K. , Kvarnemo, C. , André, C. & Panova, M. (2016). Non‐random paternity of offspring in a highly promiscuous marine snail suggests postcopulatory sexual selection. Behavioral Ecology and Sociobiology 70, 1357–1366.

[brv70013-bib-0177] John, L. K. (1993). Alternative reproductive tactics in male eastern gray squirrels: ‘making the best of a bad job’. Behavioral Ecology 4, 165–171.

[brv70013-bib-0178] Johns, J. L. , Roberts, J. A. , Clark, D. L. & Uetz, G. W. (2009). Love bites: male fang use during coercive mating in wolf spiders. Behavioral Ecology and Sociobiology 64, 13–18.

[brv70013-bib-0179] Johnstone, R. A. & Keller, L. (2000). How males can gain by harming their mates: sexual conflict, seminal toxins, and the cost of mating. The American Naturalist 156, 368–377. 10.1086/303392 29592138

[brv70013-bib-0180] Kalbitzer, U. , Bergstrom, M. L. , Carnegie, S. D. , Wikberg, E. C. , Kawamura, S. , Campos, F. A. , Jack, K. M. & Fedigan, L. M. (2017). Female sociality and sexual conflict shape offspring survival in a Neotropical primate. Proceedings of the National Academy of Sciences 114, 1892–1897. 10.1073/pnas.1608625114 PMC5338379 28167774

[brv70013-bib-0181] Kalichman, S. C. , Williams, E. A. , Cherry, C. , Belcher, L. & Nachimson, D. (1998). Sexual coercion, domestic violence, and negotiating condom use among low‐income African American women. Journal of Women's Health 7, 371–378. 10.1089/jwh.1998.7.371 9580917

[brv70013-bib-0182] Kanin, E. J. (1985). Date rapists: differential sexual socialization and relative deprivation. Archives of Sexual Behavior 14, 219–231. 4004546 10.1007/BF01542105

[brv70013-bib-0183] Kappeler, P. M. , Huchard, E. , Baniel, A. , Canteloup, C. , Charpentier, M. J. E. , Cheng, L. , Davidian, E. , Duboscq, J. , Fichtel, C. , Hemelrijk, C. K. , Höner, O. P. , Koren, L. , Micheletta, J. , Prox, L. , Saccà, T. , Seex, L. , Smit, N. , Surbeck, M. , van de Waal, E. & Girard‐Buttoz, C. (2022). Sex and dominance: how to assess and interpret intersexual dominance relationships in mammalian societies. Frontiers in Ecology and Evolution 10, 918773. 10.3389/fevo.2022.918773.

[brv70013-bib-0184] Killen, S. S. , Croft, D. P. , Salin, K. & Darden, S. K. (2016). Male sexually coercive behaviour drives increased swimming efficiency in female guppies. Functional Ecology 30, 576–583. 27478292 10.1111/1365-2435.12527 PMC4949636

[brv70013-bib-0185] Kimura, K. & Chiba, S. (2015). The direct cost of traumatic secretion transfer in hermaphroditic land snails: individuals stabbed with a love dart decrease lifetime fecundity. Proceedings of the Royal Society B: Biological Sciences 282, 20143063. 10.1098/rspb.2014.3063 PMC4375876 25761713

[brv70013-bib-0186] Kirkpatrick, J. F. & Turner, J. W. (1991). Changes in herd stallions among feral horse bands and the absence of forced copulation and induced abortion. Behavioral Ecology and Sociobiology 29, 217–219.

[brv70013-bib-0187] Kitchen, D. M. , Beehner, J. C. , Bergman, T. J. , Cheney, D. L. , Crockford, C. , Engh, A. L. , Fischer, J. , Seyfarth, R. M. & Wittig, R. M. (2009). The causes and consequences of male aggression directed at female chacma baboons. In Sexual Coercion in Primates and Humans: An Evolutionary Perspective on Male Aggression against Females (eds M. N. Muller and R. W. Wrangham ), pp. 128–156. Harvard University Press, Cambridge.

[brv70013-bib-0188] Knott, C. D. (2009). Orangutans: sexual coercion without sexual violence. In Sexual Coercion in Primates: An Evolutionary Perspective on Male Aggression against Females (eds M. N. Muller and R. W. Wrangham ), pp. 81–111. Harvard University Press, Cambridge.

[brv70013-bib-0190] Knott, C. D. & Kahlenberg, S. (2007). Orangutans in perspective: forced copulations and female mating resistance. In Primates in Perspective (eds S. Bearder , C. J. Campbell , A. Fuentes , K. C. MacKinnon and M. Panger ), pp. 290–305. Oxford University Press, Oxford.

[brv70013-bib-0189] Knott, C. D. , Emery Thompson, M. , Stumpf, R. M. & McIntyre, M. H. (2010). Female reproductive strategies in orangutans, evidence for female choice and counterstrategies to infanticide in a species with frequent sexual coercion. Proceedings of the Royal Society B: Biological Sciences 277, 105–113. 10.1098/rspb.2009.1552 PMC2842634 19812079

[brv70013-bib-0191] Koenig, A. , Miles, A. , Riaz, D.‐E.‐A. & Borries, C. (2022). Intersexual agonism in gray langurs reflects male dominance and feeding competition. Frontiers in Ecology and Evolution 10, 860437.

[brv70013-bib-0192] Kokko, H. & Brooks, R. (2003). Sexy to die for? Sexual selection and the risk of extinction. Annales Zoologici Fennici 40, 207–219.

[brv70013-bib-0193] Koss, M. P. (1993). Rape: scope, impact, interventions, and public policy responses. American Psychologist 48, 1062–1069. 8256879

[brv70013-bib-0194] Kuester, J. & Paul, A. (1992). Influence of male competition and female mate choice on male mating success in barbary macaques (*Macaca Sylvanus*). Behaviour 120, 192–216.

[brv70013-bib-0195] Kunz, J. A. , Duvot, G. J. , van Noordwijk, M. A. , Willems, E. P. , Townsend, M. , Mardianah, N. , Utami Atmoko, S. S. , Vogel, E. R. , Nugraha, T. P. , Heistermann, M. , Agil, M. , Weingrill, T. & van Schaik, C. P. (2021 *a*). The cost of associating with males for Bornean and Sumatran female orangutans: a hidden form of sexual conflict? Behavioral Ecology and Sociobiology 75(6), 1–22. 10.1007/s00265-020-02948-4 PMC7773621 33408436

[brv70013-bib-0196] Kunz, J. A. , Duvot, G. J. , Willems, E. P. , Stickelberger, J. , Spillmann, B. , Utami Atmoko, S. S. , van Noordwijk, M. A. & van Schaik, C. P. (2021 *b*). The context of sexual coercion in orang‐utans: when do male and female mating interests collide? Animal Behaviour 182, 67–90.

[brv70013-bib-0197] Kwan, L. , Cheng, Y. Y. , Rodd, F. H. & Rowe, L. (2013). Sexual conflict and the function of genitalic claws in guppies (*Poecilia reticulata*). Biology Letters 9, 20130267. 23883572 10.1098/rsbl.2013.0267 PMC3971666

[brv70013-bib-0199] Lalumière, M. L. & Lalumiere, M. L. (2005). The Causes of Rape: Understanding Individual Differences in Male Propensity for Sexual Aggression. American Psychological Association Washington, Washington.

[brv70013-bib-0200] Lalumière, M. L. & Quinsey, V. L. (1996). Sexual deviance, antisociality, mating effort, and the use of sexually coercive behaviors. Personality and Individual Differences 21, 33–48.

[brv70013-bib-0198] Lalumière, M. L. , Chalmers, L. J. , Quinsey, V. L. & Seto, M. C. (1996). A test of the mate deprivation hypothesis of sexual coercion. Ethology and Sociobiology 17, 299–318.

[brv70013-bib-0201] Lange, R. , Reinhardt, K. , Michiels, N. K. & Anthes, N. (2013). Functions, diversity, and evolution of traumatic mating. Biological Reviews 88, 585–601. 23347274 10.1111/brv.12018

[brv70013-bib-0135] Le Galliard, J.‐F. , Fitze, P. S. , Ferrière, R. & Clobert, J. (2005). Sex ratio bias, male aggression, and population collapse in lizards. Proceedings of the National Academy of Sciences 102, 18231–18236. 10.1073/pnas.0505172102 PMC1312374 16322105

[brv70013-bib-0302] le Roux, A. , Snyder‐Mackler, N. , Roberts, E. K. , Beehner, J. C. & Bergman, T. J. (2013). Evidence for tactical concealment in a wild primate. Nature Communications 4, 1462. 10.1038/ncomms2468 23403563

[brv70013-bib-0202] Lee, P. L. M. & Hays, G. C. (2004). Polyandry in a marine turtle: females make the best of a bad job. Proceedings of the National Academy of Sciences 101, 6530–6535. 10.1073/pnas.0307982101 PMC404079 15096623

[brv70013-bib-0203] Lessells, C. M. (2006). The evolutionary outcome of sexual conflict. Philosophical Transactions of the Royal Society B: Biological Sciences 361, 301–317. 10.1098/rstb.2005.1795 PMC1569608 16612889

[brv70013-bib-0204] Link, A. , Di Fiore, A. & Spehar, S. N. (2009). Female‐directed aggression and social control in spider monkeys. In Sexual Coercion in Primates and Humans: An Evolutionary Perspective on Male Aggression against Females (eds M. N. Muller and R. W. Wrangham ), pp. 157–183. Harvard University Press, Cambridge.

[brv70013-bib-0205] Linklater, W. L. , Cameron, E. Z. , Minot, E. O. & Stafford, K. J. (1999). Stallion harassment and the mating system of horses. Animal Behaviour 58, 295–306. 10458881 10.1006/anbe.1999.1155

[brv70013-bib-0206] Lötter, T. K. & Pillay, N. (2012). Social interactions associated with reproduction in the bushveld gerbil *Gerbilliscus leucogaster* . Acta Theriologica 57, 29–39.

[brv70013-bib-0207] Low, M. (2005). Female resistance and male force: context and patterns of copulation in the New Zealand stitchbird *Notiomystis cincta* . Journal of Avian Biology 36, 436–448.

[brv70013-bib-0208] Lowe, A. E. , Hobaiter, C. & Newton‐Fisher, N. E. (2019). Countering infanticide: chimpanzee mothers are sensitive to the relative risks posed by males on differing rank trajectories. American Journal of Physical Anthropology 168, 3–9. 10.1002/ajpa.23723 30302748

[brv70013-bib-0209] Loy, J. & Loy, K. (1977). Sexual harassment among captive patas monkeys (*Erythrocebus patas*). Primates 18, 691–699.

[brv70013-bib-0210] Lukas, D. & Huchard, E. (2014). The evolution of infanticide by males in mammalian societies. Science 346, 841–844. 25395534 10.1126/science.1257226

[brv70013-bib-0211] Lung, O. , Tram, U. , Finnerty, C. M. , Eipper‐Mains, M. A. , Kalb, J. M. & Wolfner, M. F. (2002). The *Drosophila melanogaster* seminal fluid protein Acp62F is a protease inhibitor that is toxic upon ectopic expression. Genetics 160, 211–224. 11805057 10.1093/genetics/160.1.211 PMC1461949

[brv70013-bib-0212] Magurran, A. & Ojanguren, A. (2007). Male harassment reduces short‐term female fitness in guppies. Behaviour 144, 503–514.

[brv70013-bib-0213] Magurran, A. E. & Seghers, B. H. (1997). A cost of sexual harassment in the guppy, *Poecilia reticulata* . Proceedings of the Royal Society of London. Series B: Biological Sciences 258, 89–92.

[brv70013-bib-0214] Manguette, M. L. , Robbins, A. M. , Breuer, T. , Stokes, E. J. , Parnell, R. J. & Robbins, M. M. (2019). Intersexual conflict influences female reproductive success in a female‐dispersing primate. Behavioral Ecology and Sociobiology 73, 118.

[brv70013-bib-0215] Martin, A. R. & da Silva, V. M. F. (2004). River dolphins and flooded forest: seasonal habitat use and sexual segregation of botos (*Inia geoffrensis*) in an extreme cetacean environment. Journal of Zoology 263, 295–305.

[brv70013-bib-0216] Martin, R. G. (1975). Sexual and aggressive behavior, density and social structure in a natural population of mosquitofish, *Gambusia affinis holbrooki* . Copeia 1975, 445–454.

[brv70013-bib-0217] Maynard, M. & Hanmer, J. (1987). Women, Violence and Social Control. Palgrave Macmillan, London.

[brv70013-bib-0218] McKibbin, W. F. , Shackelford, T. K. , Goetz, A. T. & Starratt, V. G. (2008). Why do men rape? An evolutionary psychological perspective. Review of General Psychology 12, 86–97.

[brv70013-bib-0219] McKinney, F. & Evarts, S. (1998). Sexual coercion in waterfowl and other birds. Ornithological Monographs 1997, 163–195.

[brv70013-bib-0220] McKinney, F. & Stolen, P. (1982). Extra‐pair‐bond courtship and forced copulation among captive green‐winged teal (*Anas crecca carolinensis*). Animal Behaviour 30, 461–474.

[brv70013-bib-0221] McLain, D. K. & Pratt, A. E. (1999). The cost of sexual coercion and heterospecific sexual harassment on the fecundity of a host‐specific, seed‐eating insect (*Neacoryphus bicrucis*). Behavioral Ecology and Sociobiology 46, 164–170.

[brv70013-bib-0222] McLaughlin, H. , Uggen, C. & Blackstone, A. (2012). Sexual harassment, workplace authority, and the paradox of power. American Sociological Review 77, 625–647. 23329855 10.1177/0003122412451728 PMC3544188

[brv70013-bib-0223] McMahon, C. R. & Bradshaw, C. J. A. (2004). Harem choice and breeding experience of female southern elephant seals influence offspring survival. Behavioral Ecology and Sociobiology 55, 349–362.

[brv70013-bib-0224] McRae, S. B. & Kovacs, K. M. (1994). Paternity exclusion by DNA fingerprinting, and mate guarding in the hooded seal *Cystophora cristata* . Molecular Ecology 3, 101–107. 8019687 10.1111/j.1365-294x.1994.tb00110.x

[brv70013-bib-0225] Miller, E. , Decker, M. R. , Reed, E. , Raj, A. , Hathaway, J. E. & Silverman, J. G. (2007). Male partner pregnancy‐promoting behaviors and adolescent partner violence: findings from a qualitative study with adolescent females. Ambulatory Pediatrics 7, 360–366. 17870644 10.1016/j.ambp.2007.05.007

[brv70013-bib-0226] Mineau, P. , McKinney, F. & Derrickson, S. R. (1983). Forced copulation in waterfowl. Behaviour 86, 250–293.

[brv70013-bib-0227] Mitani, J. C. (1985). Mating behaviour of male orangutans in the Kutai game reserve, Indonesia. Animal Behaviour 33, 392–402.

[brv70013-bib-0228] Moldowan, P. D. , Brooks, R. J. & Litzgus, J. D. (2020 *a*). Demographics of injuries indicate sexual coercion in a population of painted turtles (*Chrysemys picta*). Canadian Journal of Zoology 98, 269–278.

[brv70013-bib-0229] Moldowan, P. D. , Brooks, R. J. & Litzgus, J. D. (2020 *b*). Sex, shells, and weaponry: coercive reproductive tactics in the painted turtle, Chrysemys picta. Behavioral Ecology and Sociobiology 74, 142.

[brv70013-bib-0230] Møller, A. P. (1990). Deceptive use of alarm calls by male swallows, *Hirundo rustica*: a new paternity guard. Behavioral Ecology 1, 1–6.

[brv70013-bib-0231] Møller, A. P. (2004). Rapid temporal change in frequency of infanticide in a passerine bird associated with change in population density and body condition. Behavioral Ecology 15, 462–468.

[brv70013-bib-0232] Moore, A. M. , Frohwirth, L. & Miller, E. (2010). Male reproductive control of women who have experienced intimate partner violence in the United States. Social Science & Medicine 70, 1737–1744. 20359808 10.1016/j.socscimed.2010.02.009

[brv70013-bib-0233] Moreira, P. L. & Birkhead, T. R. (2004). Copulatory plug displacement and prolonged copulation in the Iberian rock lizard (*Lacerta monticola*). Behavioral Ecology and Sociobiology 56, 290–297.

[brv70013-bib-0234] Morelli, T. L. , King, S. J. , Pochron, S. T. & Wright, P. C. (2009). The rules of disengagement: takeovers, infanticide, and dispersal in a rainforest lemur, *Propithecus edwardsi* . Behaviour 146, 499–523.

[brv70013-bib-0235] Morris, M. R. , Rios‐Cardenas, O. & Darrah, A. (2008). Male mating tactics in the northern mountain swordtail fish (*Xiphophorus nezahualcoyotl*): coaxing and coercing females to mate. Ethology 114, 977–988.

[brv70013-bib-0236] Mouginot, P. , Prügel, J. , Thom, U. , Steinhoff, P. O. M. , Kupryjanowicz, J. & Uhl, G. (2015). Securing paternity by mutilating female genitalia in spiders. Current Biology 25, 2980–2984. 26549254 10.1016/j.cub.2015.09.074

[brv70013-bib-0241] Muller, M. N. & Wrangham, R. W. (eds) (2009). Sexual Coercion in Primates and Humans: An Evolutionary Perspective on Male Aggression against Females. Harvard University Press, Cambridge.

[brv70013-bib-0238] Muller, M. N. , Kahlenberg, S. M. & Wrangham, R. W. (2009 *a*). Male aggression against females and sexual coercion in chimpanzees. In Sexual Coercion in Primates and Humans: An Evolutionary Perspective on Male Aggression against Females (eds M. N. Muller and R. W. Wrangham ), pp. 184–217. Harvard University Press, Cambridge.

[brv70013-bib-0239] Muller, M. N. , Kahlenberg, S. M. & Wrangham, R. W. (2009 *b*). Male aggression and sexual coercion of females in primates. In Sexual Coercion in Primates and Humans: An Evolutionary Perspective on Male Aggression against Females (eds M. N. Muller and R. W. Wrangham ), pp. 3–22. Harvard University Press, Cambridge.

[brv70013-bib-0237] Muller, M. N. , Kahlenberg, S. M. , Emery Thompson, M. & Wrangham, R. W. (2007). Male coercion and the costs of promiscuous mating for female chimpanzees. Proceedings of the Royal Society B: Biological Sciences 274, 1009–1014. 10.1098/rspb.2006.0206 PMC2141672 17264062

[brv70013-bib-0240] Muller, M. N. , Sabbi, K. H. , Emery Thompson, M. , Enigk, D. K. , Hagberg, L. , Machanda, Z. P. , Menante, A. , Otali, E. & Wrangham, R. W. (2024). Age‐related reproductive effort in male chimpanzees: terminal investment or alternative tactics? Animal Behaviour 213, 11–21. 39007109 10.1016/j.anbehav.2024.04.002 PMC11238624

[brv70013-bib-0242] Muniz, D. G. , Baena, M. L. , Macías‐Ordóñez, R. & Machado, G. (2018). Males, but not females, perform strategic mate searching movements between host plants in a leaf beetle with scramble competition polygyny. Ecology and Evolution 8, 5828–5836. 29938096 10.1002/ece3.4121 PMC6009763

[brv70013-bib-0243] Nakata, K. (2016). Female genital mutilation and monandry in an orb‐web spider. Biology Letters 12, 20150912. 26911338 10.1098/rsbl.2015.0912 PMC4780549

[brv70013-bib-0244] Nefdt, R. J. C. (1995). Disruptions of matings, harassment and lek‐breeding in Kafue lechwe antelope. Animal Behaviour 49, 419–429.

[brv70013-bib-0245] Nefdt, R. J. C. & Thirgood, S. J. (1997). Lekking, resource defense, and harassment in two subspecies of lechwe antelope. Behavioral Ecology 8, 1–9.

[brv70013-bib-0246] Nikolajski, C. , Miller, E. , McCauley, H. L. , Akers, A. , Schwarz, E. B. , Freedman, L. , Steinberg, J. , Ibrahim, S. & Borrero, S. (2015). Race and reproductive coercion: a qualitative assessment. Women's Health Issues 25, 216–223. 25748823 10.1016/j.whi.2014.12.004 PMC4430345

[brv70013-bib-0247] Nishida, T. (1990). The Chimpanzees of the Mahale Mountains: Sexual and Life History Strategies. University of Tokyo Press, Tokyo.

[brv70013-bib-0248] Nishida, T. (2003). Harassment of mature female chimpanzees by young males in the Mahale mountains. International Journal of Primatology 24, 503–514.

[brv70013-bib-0249] Nishino, H. , Morimoto, K. , Kuroda, K. & Takami, Y. (2024). Male mate guarding in a polyandrous and sexually cannibalistic praying mantid. Behavioral Ecology and Sociobiology 78, 82.

[brv70013-bib-0250] Nordell, S. E. (1994). Observations of the mating behavior and dentition of the round stingray, *Urolophus halleri* . Environmental Biology of Fishes 39, 219–229.

[brv70013-bib-0251] Novak, S. A. & Hatch, M. A. (2009). Intimate wounds: craniofacial trauma in women and female chimpanzees. In Sexual Coercion in Primates and Humans: An Evolutionary Perspective on Male Aggression against Females (eds M. N. Muller and R. W. Wrangham ), pp. 322–345. Harvard University Press, Cambridge.

[brv70013-bib-0252] Nuñez, C. L. , Grote, M. N. , Wechsler, M. , Allen‐Blevins, C. R. & Hinde, K. (2015). Offspring of primiparous mothers do not experience greater mortality or poorer growth: revisiting the conventional wisdom with archival records of rhesus macaques. American Journal of Primatology 77, 963–973. 26031808 10.1002/ajp.22426 PMC4666832

[brv70013-bib-0253] Nunn, C. L. (1999). The evolution of exaggerated sexual swellings in primates and the graded‐signal hypothesis. Animal Behaviour 58, 229–246. 10458874 10.1006/anbe.1999.1159

[brv70013-bib-0254] Nunn, C. L. (2003). Behavioural defences against sexually transmitted diseases in primates. Animal Behaviour 66, 37–48.

[brv70013-bib-0256] O'Donohue, W. , Downs, K. & Yeater, E. A. (1998). Sexual harassment: a review of the literature. Aggression and Violent Behavior 3, 11–128.

[brv70013-bib-0255] Odendaal, F. J. , Turchin, P. & Stermitz, F. R. (1989). Influence of host‐plant density and male harassment on the distribution of female *Euphydryas anicia* (Nymphalidae). Oecologia 78, 283–288. 28312370 10.1007/BF00377167

[brv70013-bib-0257] Oleinichenko, V. Y. (2012). Behavioral interactions of adult females of the common shrew (*Sorex araneus*) with conspecifics on familiar territory. Biology Bulletin 39, 351–359. 22988759

[brv70013-bib-0258] Ollo‐López, A. & Nuñez, I. (2018). Exploring the organizational drivers of sexual harassment: empowered jobs against isolation and tolerant climates. Employee Relations 40, 174–192.

[brv70013-bib-0259] Olsson, M. (1995). Forced copulation and costly female resistance behavior in the Lake Eyre dragon, *Ctenophorus maculosus* . Herpetologica 51, 19–24.

[brv70013-bib-0260] Omland, K. E. (1996). Female mallard mating preferences for multiple male ornaments. Behavioral Ecology and Sociobiology 39, 353–360.

[brv70013-bib-0261] Opie, C. , Atkinson, Q. D. , Dunbar, R. I. M. & Shultz, S. (2013). Male infanticide leads to social monogamy in primates. Proceedings of the National Academy of Sciences 110, 13328–13332. 10.1073/pnas.1307903110 PMC3746880 23898180

[brv70013-bib-0262] Packer, C. & Pusey, A. E. (1983). Adaptations of female lions to infanticide by incoming males. The American Naturalist 121, 716–728.

[brv70013-bib-0263] Packer, C. , Scheel, D. & Pusey, A. E. (1990). Why lions form groups: food is not enough. The American Naturalist 136, 1–19.

[brv70013-bib-0264] Palombit, R. (2009). ‘Friendship’ with males: a female counterstrategy to infanticide in chacma baboons of the Okavango Delta. In Sexual Coercion in Primates and Humans: An Evolutionary Perspective on Male Aggression against Females (eds M. N. Muller and R. W. Wrangham ), pp. 377–409. Harvard University Press, Cambridge.

[brv70013-bib-0265] Palombit, R. A. (2012). Infanticide: male strategies and female counterstrategies. In The Evolution of Primate Societies (eds J. C. Mitani , J. Call , P. M. Kappeler , R. A. Palombit and J. B. Silk ), pp. 432–468. University of Chicago Press, Chicago.

[brv70013-bib-0266] Palombit, R. A. (2015). Infanticide as sexual conflict: coevolution of male strategies and female counterstrategies. Cold Spring Harbor Perspectives in Biology 7, a017640. 25986557 10.1101/cshperspect.a017640 PMC4448612

[brv70013-bib-0267] Paoli, T. (2009). The absence of sexual coercion in bonobos. In Sexual Coercion in Primates and Humans: An Evolutionary Perspective on Male Aggression against Females (eds M. N. Muller and R. W. Wrangham ), pp. 410–423. Harvard University Press, Cambridge.

[brv70013-bib-0268] Parker, G. & Partridge, L. (1998). Sexual conflict and speciation. Philosophical Transactions of the Royal Society of London. Series B: Biological Sciences 353, 261–274. 9533125 10.1098/rstb.1998.0208 PMC1692203

[brv70013-bib-0269] Parker, G. A. (1970). Sperm competition and its evolutionary consequences in the insects. Biological Reviews 45, 525–567.

[brv70013-bib-0270] Parker, G. A. (1979). Sexual selection and sexual conflict. In Sexual Selection and Reproductive Competition in Insects (eds M. S. Blum and N. A. Blum ), pp. 123–166. Academic Press, New York.

[brv70013-bib-0271] Parkes, A. S. & Bruce, H. M. (1961). Olfactory stimuli in mammalian reproduction. Science 134, 1049–1054. 14483939 10.1126/science.134.3485.1049

[brv70013-bib-0272] Pavé, R. , Kowalewski, M. M. , Garber, P. A. , Zunino, G. E. , Fernandez, V. A. & Peker, S. M. (2012). Infant mortality in black‐and‐gold howlers (*Alouatta caraya*) living in a flooded forest in northeastern Argentina. International Journal of Primatology 33, 937–957.

[brv70013-bib-0273] Penteriani, V. , Kojola, I. , Heikkinen, S. , Find'o, S. , Skuban, M. , Fedorca, A. , García‐Sánchez, P. , Fedorca, M. , Zarzo‐Arias, A. , Balbontín, J. & Delgado, M. d. M. (2024). Livin’ on the edge: reducing infanticide risk by maintaining proximity to potentially less infanticidal males. Animal Behaviour 210, 63–71.

[brv70013-bib-0274] Pereira, M. E. (1983). Abortion following the immigration of an adult male baboon (*Papio cynocephalus*). American Journal of Primatology 4, 93–98. 31991967 10.1002/ajp.1350040109

[brv70013-bib-0275] Perry, S. (2012). The behavior of wild white‐faced capuchins: demography, life history, social relationships, and communication. In Advances in the Study of Behavior (eds H. J. Brockmann , T. J. Roper , M. Naguib , J. C. Mitani and L. W. Simmons ), pp. 135–181. Academic Press, San Diego.

[brv70013-bib-0276] Petrulis, A. (2013). Chemosignals, hormones and mammalian reproduction. Hormones and Behavior 63, 723–741. 23545474 10.1016/j.yhbeh.2013.03.011 PMC3667964

[brv70013-bib-0277] Pilastro, A. , Benetton, S. & Bisazza, A. (2003). Female aggregation and male competition reduce costs of sexual harassment in the mosquitofish *Gambusia holbrooki* . Animal Behaviour 65, 1161–1167.

[brv70013-bib-0278] Pistorius, P. A. , Bester, M. N. , Kirkman, S. P. & Taylor, F. E. (2001). Pup mortality in southern elephant seals at Marion Island. Polar Biology 24, 828–831.

[brv70013-bib-0279] Pizzari, T. & Birkhead, T. R. (2000). Female feral fowl eject sperm of subdominant males. Nature 405, 787–789. 10866198 10.1038/35015558

[brv70013-bib-0280] Plath, M. , Parzefall, J. & Schlupp, I. (2003). The role of sexual harassment in cave and surface dwelling populations of the Atlantic molly, *Poecilia mexicana* (*Poeciliidae, Teleostei*). Behavioral Ecology and Sociobiology 54, 303–309.

[brv70013-bib-0281] Pluháček, J. & Bartoš, L. (2000). Male infanticide in captive plains zebra, *Equus burchelli* . Animal Behaviour 59, 689–694. 10792924 10.1006/anbe.1999.1371

[brv70013-bib-0282] Poole, J. H. (1989). Mate guarding, reproductive success and female choice in African elephants. Animal Behaviour 37, 842–849.

[brv70013-bib-0283] Pradhan, G. R. & van Schaik, C. P. (2009). Why do females find ornaments attractive? The coercion avoidance hypothesis. Biological Journal of the Linnean Society 11, 372–382.

[brv70013-bib-0284] Pratt, H. L. Jr. (1979). Reproduction in the blue shark, *Prionace glauca* . Fishery Bulletin 77, 445–470.

[brv70013-bib-0285] Preston, B. T. , Stevenson, I. R. , Pemberton, J. M. , Coltman, D. W. & Wilson, K. (2005). Male mate choice influences female promiscuity in Soay sheep. Proceedings of the Royal Society B: Biological Sciences 272, 365–373. 10.1098/rspb.2004.2977 PMC1634988 15734690

[brv70013-bib-0286] Prosen, E. D. , Jaeger, R. G. & Lee, D. R. (2004). Sexual coercion in a territorial salamander: females punish socially polygynous male partners. Animal Behaviour 67, 85–92.

[brv70013-bib-0287] Réale, D. , Boussès, P. & Chapuis, J.‐L. (1996). Female‐biased mortality induced by male sexual harassment in a feral sheep population. Canadian Journal of Zoology 74, 1812–1818.

[brv70013-bib-0288] Reddy, R. B. , Langergraber, K. E. , Sandel, A. A. , Vigilant, L. & Mitani, J. C. (2021). The development of affiliative and coercive reproductive tactics in male chimpanzees. Proceedings of the Royal Society B: Biological Sciences 288, 20202679. 10.1098/rspb.2020.2679 PMC7892417 33402074

[brv70013-bib-0289] Reinhardt, K. , Anthes, N. & Lange, R. (2015). Copulatory wounding and traumatic insemination. Cold Spring Harbor Perspectives in Biology 7, a017582. 25877218 10.1101/cshperspect.a017582 PMC4448625

[brv70013-bib-0290] Reyer, H. , Frei, G. & Som, C. (1999). Cryptic female choice: frogs reduce clutch size when amplexed by undesired males. Proceedings of the Royal Society of London. Series B: Biological Sciences 266, 2101–2107. 10.1098/rspb.1999.0894 PMC1690325 10902545

[brv70013-bib-0292] Robbins, A. M. , Stoinski, T. S. , Fawcett, K. A. & Robbins, M. M. (2009 *b*). Socioecological influences on the dispersal of female mountain gorillas—evidence of a second folivore paradox. Behavioral Ecology and Sociobiology 63, 477–489.

[brv70013-bib-0291] Robbins, A. M. , Stoinski, T. , Fawcett, K. & Robbins, M. M. (2009 *a*). Leave or conceive: natal dispersal and philopatry of female mountain gorillas in the Virunga volcano region. Animal Behaviour 77, 831–838.

[brv70013-bib-0293] Robbins, M. & Sawyer, S. (2007). Intergroup encounters in mountain gorillas of Bwindi impenetrable National Park, Uganda. Behaviour 144, 1497–1519.

[brv70013-bib-0294] Robbins, M. M. (2009). Male aggression against females in mountain gorillas: courtship or coercion. In Sexual Coercion in Primates: An Evolutionary Perspective on Male Aggression against Females (eds M. N. Muller and R. W. Wrangham ), pp. 112–127. Harvard University Press, Cambridge.

[brv70013-bib-0295] Roberts, E. K. , Lu, A. , Bergman, T. J. & Beehner, J. C. (2012). A Bruce effect in wild geladas. Science 335, 1222–1225. 22362878 10.1126/science.1213600

[brv70013-bib-0296] Roberts, J. D. & Byrne, P. G. (2011). Polyandry, sperm competition, and the evolution of anuran amphibians. In Advances in the Study of Behavior (eds H. J. Brockmann , T. J. Roper , M. Naguib , J. C. Mitani and L. W. Simmons ), pp. 1–53. Academic Press, San Diego.

[brv70013-bib-0297] Robertson, H. M. (1985). Female dimorphism and mating behaviour in a damselfly, *Ischnura ramburi*: females mimicking males. Animal Behaviour 33, 805–809.

[brv70013-bib-0298] Roesli, M. & Reyer, H.‐U. (2000). Male vocalization and female choice in the hybridogenetic *Rana lessonae/Rana esculenta* complex. Animal Behaviour 60, 745–755. 11124872 10.1006/anbe.2000.1519

[brv70013-bib-0299] Rohwer, S. , Harris, R. B. & Walsh, H. E. (2014). Rape and the prevalence of hybrids in broadly sympatric species: a case study using albatrosses. PeerJ 2, e409. 24949232 10.7717/peerj.409 PMC4060039

[brv70013-bib-0300] Rönn, J. , Katvala, M. & Arnqvist, G. (2007). Coevolution between harmful male genitalia and female resistance in seed beetles. Proceedings of the National Academy of Sciences 104, 10921–10925. 10.1073/pnas.0701170104 PMC1904142 17573531

[brv70013-bib-0301] Rossi, B. H. , Nonacs, P. & Pitts‐Singer, T. L. (2010). Sexual harassment by males reduces female fecundity in the alfalfa leafcutting bee, *Megachile rotundata* . Animal Behaviour 79, 165–171.

[brv70013-bib-0303] Rowe, L. (1992). Convenience polyandry in a water strider: foraging conflicts and female control of copulation frequency and guarding duration. Animal Behaviour 44, 189–202.

[brv70013-bib-0304] Rowe, L. (1994). The costs of mating and mate choice in water striders. Animal Behaviour 48, 1049–1056.

[brv70013-bib-0305] Rowe, L. , Arnqvist, G. , Sih, A. & Krupa, J. (1994). Sexual conflict and the evolutionary ecology of mating patterns: water striders as a model system. Trends in Ecology & Evolution 9, 289–293. 21236857 10.1016/0169-5347(94)90032-9

[brv70013-bib-0306] Rubenstein, D. (1994). The ecology of female social behavior in horses, zebras, and asses. In Animal Societies: Individuals, Interactions, and Organization. Kyoto University Press, Kyoto.

[brv70013-bib-0307] Rubenstein, D. I. (1986). Ecology and sociality in horses and zebras. In Ecological Aspects of Social Evolution (eds D. I. Rubenstein and W. R. Wrangham ), pp. 282–302. Princeton University Press, Princeton.

[brv70013-bib-0308] Rudd, C. D. (1994). Sexual behaviour of male and female tammar wallabies (*Macropus eugenii*) at post‐partum oestrus. Journal of Zoology 232, 151–162.

[brv70013-bib-0309] Rudran, R. (1973). Adult male replacement in one‐male troops of purple‐faced langurs (*Presbytis senex senex*) and its effect on population structure. Folia Primatologica 19, 166–192. 10.1159/000155537 4201908

[brv70013-bib-0310] Rueda‐Solano, L. A. , Vargas‐Salinas, F. , Pérez‐González, J. L. , Sánchez‐Ferreira, A. , Ramírez‐Guerra, A. , Navas, C. A. & Crawford, A. J. (2022). Mate‐guarding behaviour in anurans: intrasexual selection and the evolution of prolonged amplexus in the harlequin toad *Atelopus laetissimus* . Animal Behaviour 185, 127–142.

[brv70013-bib-0311] Sargent, R. C. , Gross, M. R. & Van Den Berghe, E. P. (1986). Male mate choice in fishes. Animal Behaviour 34, 545–550.

[brv70013-bib-0312] Sato, T. , Sugiyama, T. & Sekijima, T. (2023). Mating in the cold. Prolonged sperm storage provides opportunities for forced copulation by male bats during winter. Frontiers in Physiology 14, 1241470. 37745243 10.3389/fphys.2023.1241470 PMC10511888

[brv70013-bib-0313] Saunders, F. C. , McElligott, A. G. , Safi, K. & Hayden, T. J. (2005). Mating tactics of male feral goats (*Capra hircus*): risks and benefits. Acta Ethologica 8, 103–110.

[brv70013-bib-0315] Schlegel, A. & Barry, H. (1986). The cultural consequences of female contribution to subsistence. American Anthropologist 88, 142–150.

[brv70013-bib-0316] Scott, A. M. , Susanto, T. W. , Setia, T. M. & Knott, C. D. (2023). Mother‐offspring proximity maintenance as an infanticide avoidance strategy in bornean orangutans (*Pongo pygmaeus wurmbii*). American Journal of Primatology 85, e23482. 36871268 10.1002/ajp.23482

[brv70013-bib-0317] Scott, E. M. , Mann, J. , Watson‐Capps, J. J. , Sargeant, B. L. & Connor, R. C. (2005). Aggression in bottlenose dolphins: evidence for sexual coercion, male‐male competition, and female tolerance through analysis of tooth‐rake marks and behaviour. Behaviour 142, 21–44.

[brv70013-bib-0318] Setchell, J. M. (2005). Do female mandrills prefer brightly colored males? International Journal of Primatology 26, 715–735.

[brv70013-bib-0320] Setchell, J. M. & Jean Wickings, E. (2006). Mate choice in male mandrills (*Mandrillus sphinx*). Ethology 112, 91–99.

[brv70013-bib-0319] Setchell, J. M. , Charpentier, M. & Wickings, E. J. (2005). Mate guarding and paternity in mandrills: factors influencing alpha male monopoly. Animal Behaviour 70, 1105–1120.

[brv70013-bib-0321] Setchell, J. M. , Lee, P. C. , Wickings, E. J. & Dixson, A. F. (2001). Growth and ontogeny of sexual size dimorphism in the mandrill (*Mandrillus sphinx*). American Journal of Physical Anthropology 115, 349–360. 11471133 10.1002/ajpa.1091

[brv70013-bib-0322] Shackelford, T. K. (2002). Are young women the special targets of rape‐murder? Aggressive Behavior 28, 224–232.

[brv70013-bib-0323] Shackelford, T. K. , Buss, D. M. & Weekes‐Shackelford, V. A. (2003). Wife killings committed in the context of a lovers triangle. Basic and Applied Social Psychology 25, 137–143.

[brv70013-bib-0324] Shackelford, T. K. , Goetz, A. T. , Guta, F. E. & Schmitt, D. P. (2006). Mate guarding and frequent in‐pair copulation in humans. Human Nature 17, 239–252. 26181471 10.1007/s12110-006-1007-x

[brv70013-bib-0325] Shine, R. , Langkilde, T. & Mason, R. T. (2003). Cryptic forcible insemination: male snakes exploit female physiology, anatomy, and behavior to obtain coercive matings. The American Naturalist 162, 653–667. 10.1086/378749 14618542

[brv70013-bib-0326] Shine, R. , O'Connor, D. & Mason, R. T. (2000). Sexual conflict in the snake den. Behavioral Ecology and Sociobiology 48, 392–401.

[brv70013-bib-0327] Singh, R. , Nigam, P. , Qureshi, Q. , Sankar, K. , Krausman, P. R. & Goyal, S. P. (2014). Strategy of female tigers to avoid infanticide. Current Science 107, 1595–1597.

[brv70013-bib-0328] Singh, S. , Singh, P. & Mishra, G. (2022). Sexual coercion. In Reproductive Strategies in Insects (eds Omkar and G. Mishra ), pp. 183–204. CRC Press, Boca Raton.

[brv70013-bib-0329] Smit, N. (2022). Social and Reproductive Conflicts between the Sexes in Mandrills. University of Montpellier, Montpellier.

[brv70013-bib-0330] Smit, N. (2024). Intersexual relationships in mandrills: dominance, sexual conflict and the influence of social integration. Revue de Primatologie 15, 1–12.

[brv70013-bib-0334] Smit, N. & Robbins, M. M. (2024). Female gorillas compete for food and males. Evolution and Human Behavior 45, 106611.

[brv70013-bib-0331] Smit, N. , Baniel, A. , Roura‐Torres, B. , Amblard‐Rambert, P. , Charpentier, M. J. E. & Huchard, E. (2022 *a*). Sexual coercion in a natural mandrill population. Peer Community Journal 2, e36.

[brv70013-bib-0332] Smit, N. , Dezeure, J. , Sauvadet, L. , Huchard, E. & Charpentier, M. J. E. (2023). Socially bonded females face more sexual coercion in a female‐philopatric primate. iScience 26, 107358. 37766985 10.1016/j.isci.2023.107358 PMC10520811

[brv70013-bib-0333] Smit, N. , Ngoubangoye, B. , Charpentier, M. J. E. & Huchard, E. (2022 *b*). Dynamics of intersexual dominance in a highly dimorphic primate. Frontiers in Ecology and Evolution 10, 931226.

[brv70013-bib-0335] Smith, D. W. , Metz, M. C. , Cassidy, K. A. , Stahler, E. E. , McIntyre, R. T. , Almberg, E. S. & Stahler, D. R. (2015). Infanticide in wolves: seasonality of mortalities and attacks at dens support evolution of territoriality. Journal of Mammalogy 96, 1174–1183.

[brv70013-bib-0336] Smuts, B. (1992). Male aggression against women: an evolutionary perspective. Human Nature 3, 1–44. 24222394 10.1007/BF02692265

[brv70013-bib-0337] Smuts, B. B. (1985). Sex and Friendship in Baboons. Aldine Publishing Co, Hawthorne.

[brv70013-bib-0338] Smuts, B. B. & Smuts, R. W. (1993). Male aggression and sexual coercion of females in nonhuman primates and other mammals: evidence and theoretical implications. Advances in the Study of Behavior 22, 1–63.

[brv70013-bib-0339] Snow, S. S. , Alonzo, S. H. , Servedio, M. R. & Prum, R. O. (2019). Female resistance to sexual coercion can evolve to preserve the indirect benefits of mate choice. Journal of Evolutionary Biology 32, 545–558. 30817033 10.1111/jeb.13436 PMC7045708

[brv70013-bib-0340] Soltis, J. , Mitsunaga, F. , Shimizu, K. , Yanagihara, Y. & Nozaki, M. (1997). Sexual selection in Japanese macaques I: female mate choice or male sexual coercion? Animal Behaviour 54, 725–736. 9299056 10.1006/anbe.1997.0567

[brv70013-bib-0341] Soltis, J. , Thomsen, R. , Matsubayashi, K. & Takenaka, O. (2000). Infanticide by resident males and female counter‐strategies in wild Japanese macaques (*Macaca fuscata*). Behavioral Ecology and Sociobiology 48, 195–202.

[brv70013-bib-0342] Sorenson, L. G. (1994). Forced extra‐pair copulation in the white‐cheeked pintail: male tactics and female responses. The Condor 96, 400–410.

[brv70013-bib-0343] Stacey, P. B. (1982). Female promiscuity and male reproductive success in social birds and mammals. The American Naturalist 120, 51–64.

[brv70013-bib-0344] Staedler, M. & Riedman, M. (1993). Fatal mating injuries in female sea otters (*Enhydra lutris nereis*). Mammalia 57, 135–139.

[brv70013-bib-0345] Stanko, E. A. (1990). Everyday Violence: How Women and Men Experience Sexual and Physical Danger. Pandora, London.

[brv70013-bib-0346] Stehn, R. A. & Richmond, M. E. (1975). Male‐induced pregnancy termination in the prairie vole, *Microtus ochrogaster* . Science 187, 1211–1213. 1114340 10.1126/science.1114340

[brv70013-bib-0347] Sterck, E. H. M. & Korstjens, A. H. (2000). Female dispersal and infanticide avoidance in primates. In Infanticide by Males and its Implications (eds C. P. Van Schaik and C. H. Jason ), pp. 293–321. Cambridge University Press, Cambridge.

[brv70013-bib-0348] Stieglitz, J. , Trumble, B. C. , Kaplan, H. & Gurven, M. (2018). Marital violence and fertility in a relatively egalitarian high‐fertility population. Nature Human Behaviour 2, 565–572. 10.1038/s41562-018-0391-7 PMC6499491 31058232

[brv70013-bib-0349] Stirling, I. , Andriashek, D. & Calvert, W. (1993). Habitat preferences of polar bears in the western Canadian Arctic in late winter and spring. Polar Record 29, 13–24.

[brv70013-bib-0350] Stockley, P. (2002). Sperm competition risk and male genital anatomy: comparative evidence for reduced duration of female sexual receptivity in primates with penile spines. Evolutionary Ecology 16, 123–137.

[brv70013-bib-0351] Stokes, E. J. (2004). Within‐group social relationships among females and adult males in wild western lowland gorillas (*Gorilla gorilla gorilla*). American Journal of Primatology 64, 233–246. 15470744 10.1002/ajp.20074

[brv70013-bib-0352] Stone, G. N. (1995). Female foraging responses to sexual harassment in the solitary bee *Anthophora plumipes* . Animal Behaviour 50, 405–412.

[brv70013-bib-0353] Strauss, E. D. & Holekamp, K. E. (2019). Social alliances improve rank and fitness in convention‐based societies. Proceedings of the National Academy of Sciences 116, 8919–8924. 10.1073/pnas.1810384116 PMC6500164 30858321

[brv70013-bib-0354] Stumpf, R. M. & Boesch, C. (2005). Does promiscuous mating preclude female choice? Female sexual strategies in chimpanzees (*Pan troglodytes verus*) of the Tai National Park, Côte D'ivoire. Behavioral Ecology and Sociobiology 57, 511–524.

[brv70013-bib-0355] Stumpf, R. M. & Boesch, C. (2006). The efficacy of female choice in chimpanzees of the tai Forest, Cote d'Ivoire. Behavioral Ecology and Sociobiology 60, 749–765.

[brv70013-bib-0356] Stumpf, R. M. , Martinez‐Mota, R. , Milich, K. M. , Righini, N. & Shattuck, M. R. (2011). Sexual conflict in primates. Evolutionary Anthropology: Issues, News, and Reviews 20, 62–75. 10.1002/evan.20297 22034105

[brv70013-bib-0357] Swedell, L. & Saunders, J. (2006). Infant mortality, paternity certainty, and female reproductive strategies in hamadryas baboons. In Reproduction and Fitness in Baboons: Behavioral, Ecological, and Life History Perspectives (eds L. Swedell and S. R. Leigh ), pp. 19–51. Springer, Boston, MA.

[brv70013-bib-0358] Swedell, L. & Schreier, A. (2009). Male aggression towards females in hamadryas baboons: conditioning, coercion, and control. In Sexual Coercion in Primates: An Evolutionary Perspective on Male Aggression against Females (eds M. N. Muller and R. W. Wrangham ), pp. 244–268. Harvard University Press, Cambridge.

[brv70013-bib-0359] Sztatecsny, M. , Jehle, R. , Burke, T. & Hödl, W. (2006). Female polyandry under male harassment: the case of the common toad (*Bufo bufo*). Journal of Zoology 270, 517–522.

[brv70013-bib-0360] Taborsky, M. (2008). Alternative reproductive tactics in fish. In Alternative Reproductive Tactics: An Integrative Approach (eds H. J. Brockmann , M. Taborsky and R. F. Oliveira ), pp. 251–299. Cambridge University Press, Cambridge.

[brv70013-bib-0361] Takahashi, Y. & Watanabe, M. (2010). Female reproductive success is affected by selective male harassment in the damselfly *Ischnura senegalensis* . Animal Behaviour 79, 211–216.

[brv70013-bib-0362] Takami, Y. , Sasabe, M. , Nagata, N. & Sota, T. (2008). Dual function of seminal substances for mate guarding in a ground beetle. Behavioral Ecology 19, 1173–1178.

[brv70013-bib-0363] Tarzia, L. & McKenzie, M. (2024). Reproductive coercion and abuse in intimate relationships: women's perceptions of perpetrator motivations. PLoS One 19, e0299069. 38626011 10.1371/journal.pone.0299069 PMC11020648

[brv70013-bib-0364] Tatarnic, N. J. , Cassis, G. & Siva‐Jothy, M. T. (2014). Traumatic insemination in terrestrial arthropods. Annual Review of Entomology 59, 245–261. 10.1146/annurev-ento-011613-162111 24160423

[brv70013-bib-0366] Teichroeb, J. A. & Sicotte, P. (2008). Infanticide in ursine colobus monkeys (*Colobus vellerosus*) in Ghana: new cases and a test of the existing hypotheses. Behaviour 145, 727–755.

[brv70013-bib-0365] Teichroeb, J. , Wikberg, E. & Sicotte, P. (2009). Female dispersal patterns in six groups of ursine colobus (*Colobus vellerosus*): infanticide avoidance is important. Behaviour 146, 551–582.

[brv70013-bib-0367] Thompson, M. E. (2009). In Sexual Coercion in Primates: An Evolutionary Perspective on Male Aggression against Females (eds M. N. Muller and R. W. Wrangham ), pp. 346–374. Harvard University Press, Cambridge.

[brv70013-bib-0368] Thonhauser, K. E. , Raveh, S. , Hettyey, A. , Beissmann, H. & Penn, D. J. (2013). Why do female mice mate with multiple males? Behavioral Ecology and Sociobiology 67, 1961–1970. 24273373 10.1007/s00265-013-1604-8 PMC3827896

[brv70013-bib-0369] Thornhill, R. (1980). Rape in *Panorpa* scorpionflies and a general rape hypothesis. Animal Behaviour 28, 52–59.

[brv70013-bib-0370] Thornhill, R. & Palmer, C. T. (2000). A Natural History of Rape: Biological Bases of Sexual Coercion. The MIT Press, Cambridge.

[brv70013-bib-0371] Thornhill, R. & Wilmsen Thornhill, N. (1983). Human rape: an evolutionary analysis. Ethology and Sociobiology 4, 137–173.

[brv70013-bib-0372] Tiddi, B. , Wheeler, B. C. & Heistermann, M. (2015). Female behavioral proceptivity functions as a probabilistic signal of fertility, not female quality, in a New World primate. Hormones and Behavior 73, 148–155. 26188948 10.1016/j.yhbeh.2015.07.011

[brv70013-bib-0373] Tokuyama, N. & Furuichi, T. (2016). Do friends help each other? Patterns of female coalition formation in wild bonobos at Wamba. Animal Behaviour 119, 27–35.

[brv70013-bib-0374] Towers, J. R. , Hallé, M. J. , Symonds, H. K. , Sutton, G. J. , Morton, A. B. , Spong, P. , Borrowman, J. P. & Ford, J. K. B. (2018). Infanticide in a mammal‐eating killer whale population. Scientific Reports 8, 4366. 29559642 10.1038/s41598-018-22714-x PMC5861072

[brv70013-bib-0375] Trivers, R. (1972). Parental Investment and Sexual Selection. In Sexual Selection & the Descent of Man, pp. 136–179. Aldine De Gruyter, New York.

[brv70013-bib-0376] Trumbo, S. T. (1990). Reproductive benefits of infanticide in a biparental burying beetle *Nicrophorus orbicollis* . Behavioral Ecology and Sociobiology 27, 269–273.

[brv70013-bib-0377] Trumbo, S. T. (2007). Defending young biparentally: female risk‐taking with and without a male in the burying beetle, *Nicrophorus pustulatus* . Behavioral Ecology and Sociobiology 61, 1717–1723.

[brv70013-bib-0378] Valera, F. , Hoi, H. & Krištín, A. (2003). Male shrikes punish unfaithful females. Behavioral Ecology 14, 403–408.

[brv70013-bib-0314] van Schaik, C. P. & Janson, C. H. (2000). Infanticide by Males and its Implications. Cambridge University Press, Cambridge.

[brv70013-bib-0379] Veiga, J. (2000). In Infanticide by Males and its Implications (eds C. P. van Schaik and C. H. Janson ), pp. 198–220. Cambridge University Press, Cambridge.

[brv70013-bib-0380] Waage, J. K. (1979). Dual function of the damselfly penis: sperm removal and transfer. Science 203, 916–918. 17771731 10.1126/science.203.4383.916

[brv70013-bib-0381] Walker, K. & Sleath, E. (2017). A systematic review of the current knowledge regarding revenge pornography and non‐consensual sharing of sexually explicit media. Aggression and Violent Behavior 36, 9–24.

[brv70013-bib-0382] Wallen, M. M. , Patterson, E. M. , Krzyszczyk, E. & Mann, J. (2016). The ecological costs to females in a system with allied sexual coercion. Animal Behaviour 115, 227–236.

[brv70013-bib-0383] Waterman, J. , Wolff, J. & Sherman, P. (2007). Male mating strategies in rodents. In Rodent Societies: An Ecological and Evolutionary Perspective, pp. 27–41. University of Chicago Press, Chicago.

[brv70013-bib-0385] Watson‐Capps, J. J. (2009). Evolution of sexual coercion with respect to sexual selection and sexual conflict theory. In Sexual Coercion in Primates: An Evolutionary Perspective on Male Aggression against Females (eds M. N. Muller and R. W. Wrangham ), pp. 23–41. Harvard University Press, Cambridge.

[brv70013-bib-0384] Watson, P. J. , Stallmann, R. R. & Arnqvist, G. (1998). Sexual conflict and the energetic costs of mating and mate choice in water striders. The American Naturalist 151, 46–58. 10.1086/286101 18811423

[brv70013-bib-0386] Watters, J. V. (2005). Can the alternative male tactics ‘fighter’ and ‘sneaker’ be considered ‘coercer’ and ‘cooperator’ in coho salmon? Animal Behaviour 70, 1055–1062.

[brv70013-bib-0387] Watts, D. P. (1989). Infanticide in mountain gorillas: new cases and a reconsideration of the evidence. Ethology 81, 1–18.

[brv70013-bib-0388] Watts, D. P. (1990). Ecology of gorillas and its relation to female transfer in mountain gorillas. International Journal of Primatology 11, 21–45.

[brv70013-bib-0389] Watts, D. P. (1998). Seasonality in the ecology and life histories of mountain gorillas (*Gorilla gorilla beringei*). International Journal of Primatology 19, 929–948.

[brv70013-bib-0390] Watts, D. P. (2022). Male chimpanzee sexual coercion and mating success at Ngogo. American Journal of Primatology 84, e23361. 35029301 10.1002/ajp.23361

[brv70013-bib-0392] Watts, D. P. & Pusey, A. E. (1993). Behavior of juvenile and adolescent great apes. In Juvenile Primates: Life History, Development, and Behavior (eds M. Pereira and L. Fairbanks ). University of Chicago Press, Chicago.

[brv70013-bib-0391] Watts, D. P. , Colmenares, F. & Arnold, K. (2000). In Natural Conflict Resolution (eds F. Aureli and F. B. M. De Waal ), pp. 281–301. University of California Press, Berkeley, CA.

[brv70013-bib-0393] Wearmouth, V. J. , Southall, E. J. , Morritt, D. , Thompson, R. C. , Cuthill, I. C. , Partridge, J. C. & Sims, D. W. (2012). Year‐round sexual harassment as a behavioral mediator of vertebrate population dynamics. Ecological Monographs 82, 351–366.

[brv70013-bib-0394] Westneat, D. F. & Stewart, I. R. K. (2003). Extra‐pair paternity in birds: causes, correlates, and conflict. Annual Review of Ecology, Evolution, and Systematics 34, 365–396.

[brv70013-bib-0395] Wilcox, R. S. (1984). Male copulatory guarding enhances female foraging in a water strider. Behavioral Ecology and Sociobiology 15, 171–174.

[brv70013-bib-0396] Wildermuth, H. , Jödicke, R. , Schröter, A. & Borkenstein, A. (2019). Death feigning in sexual conflict between dragonflies (Odonata): does it exist? Odonatologica 48, 211–228.

[brv70013-bib-0397] Willis, P. M. & Dill, L. M. (2007). Mate guarding in male Dall's porpoises (*Phocoenoides dalli*). Ethology 113, 587–597.

[brv70013-bib-0398] Wilson, M. & Daly, M. (2009). Coercive violence by human males against their female partners. In Sexual Coercion in Primates: An Evolutionary Perspective on Male Aggression against Females (eds M. N. Muller and R. W. Wrangham ), pp. 271–291. Harvard University Press, Cambridge.

[brv70013-bib-0399] Winkler, P. , Loch, H. & Vogel, C. (1984). Life history of hanuman langurs (*Presbytis entellus*). Folia Primatologica 43, 1–23. 10.1159/000156167 6500408

[brv70013-bib-0400] Wolf, J. B. W. , Kauermann, G. & Trillmich, F. (2005). Males in the shade: habitat use and sexual segregation in the Galápagos sea lion (*Zalophus californianus wollebaeki*). Behavioral Ecology and Sociobiology 59, 293–302.

[brv70013-bib-0401] Wolff, J. O. & Macdonald, D. W. (2004). Promiscuous females protect their offspring. Trends in Ecology & Evolution 19, 127–134. 16701243 10.1016/j.tree.2003.12.009

[brv70013-bib-0402] Wolff, J. O. & Peterson, J. A. (1998). An offspring‐defense hypothesis for territoriality in female mammals. Ethology Ecology & Evolution 10, 227–239.

[brv70013-bib-0403] World Health Organization (2012). Understanding and Addressing Violence Against Women: Sexual Violence. World Health Organization, Geneva​.

[brv70013-bib-0404] Wrangham, R. W. & Muller, M. N. (2009). Sexual coercion in humans and other primates: the road ahead. In Sexual Coercion in Primates: An Evolutionary Perspective on Male Aggression against Females (eds M. N. Muller and R. W. Wrangham ), pp. 451–468. Harvard University Press, Massachusetts.

[brv70013-bib-0405] Xiang, Z. , Yu, Y. , Yao, H. , Hu, Q. , Yang, W. & Li, M. (2022). Female countertactics to male feticide and infanticide in a multilevel primate society. Behavioral Ecology 33, 679–687.

[brv70013-bib-0406] Yamagiwa, J. , Kahekwa, J. & Basabose, A. K. (2009). Infanticide and social flexibility in the genus *Gorilla* . Primates 50, 293–303. 19688234 10.1007/s10329-009-0163-0

[brv70013-bib-0407] Yamaguchi, N. , Dugdale, H. L. & Macdonald, D. W. (2006). Female receptivity, embryonic diapause, and superfetation in the European badger (*Meles meles*): implications for the reproductive tactics of males and females. The Quarterly Review of Biology 81, 33–48. 16602273 10.1086/503923

[brv70013-bib-0408] Zeiss, C. , Martens, A. & Rolff, J. (1999). Male mate guarding increases females' predation risk? A case study on tandem oviposition in the damselfly *Coenagrion puella* (Insecta: Odonata). Canadian Journal of Zoology 77, 1013–1016.

[brv70013-bib-0409] Zhao, Q. , Borries, C. & Pan, W. (2011). Male takeover, infanticide, and female countertactics in white‐headed leaf monkeys (*Trachypithecus leucocephalus*). Behavioral Ecology and Sociobiology 65, 1535–1547.

[brv70013-bib-0410] Zinner, D. & Deschner, T. (2000). Sexual swellings in female hamadryas baboons after male take‐overs: ‘deceptive’ swellings as a possible female counter‐strategy against infanticide. American Journal of Primatology 52, 157–168. 11132110 10.1002/1098-2345(200012)52:4<157::AID-AJP1>3.0.CO;2-L

[brv70013-bib-0411] Zipple, M. N. , Grady, J. H. , Gordon, J. B. , Chow, L. D. , Archie, E. A. , Altmann, J. & Alberts, S. C. (2017). Conditional fetal and infant killing by male baboons. Proceedings of the Royal Society B: Biological Sciences 284, 20162561. 10.1098/rspb.2016.2561 PMC5310045 28100822

[brv70013-bib-0412] Zipple, M. N. , Roberts, E. K. , Alberts, S. C. & Beehner, J. C. (2019). Male‐mediated prenatal loss: functions and mechanisms. Evolutionary Anthropology: Issues, News, and Reviews 28, 114–125. 10.1002/evan.21776 PMC6548597 30953577

[brv70013-bib-0413] Zucker, E. L. & Kaplan, J. R. (1981). A reinterpretation of ‘sexual harassment’ in patas monkeys. Animal Behaviour 29, 957–958.

